# Temporal Multi-Omics Analysis of Mouse Bone Marrow Responses to Radiation Exposure

**DOI:** 10.3390/ijms27209363

**Published:** 2026-10-21

**Authors:** Ashley D. Clifton, Micah J. Jeppesen, Shohaib Shaffiey, Arpita Chatterjee, Kia T. Liermann-Wooldrik, Oluwaseun Adebisi, Simran Takkar, Naresh Mutha, Rebecca E. Oberley-Deegan, Chittibabu Guda, Massimiliano Pierobon, James W. Checco, Robert Powers

**Affiliations:** 1 Department of Chemistry, University of Nebraska-Lincoln, Lincoln, NE 68588-0304, USA; aclifton14@huskers.unl.edu (A.D.C.); mjeppesen3@huskers.unl.edu (M.J.J.); 2 School of Computing, University of Nebraska-Lincoln, Lincoln, NE 68588-0115, USA; sshaffiey2@huskers.unl.edu (S.S.); maxp@unl.edu (M.P.); 3 Department of Biochemistry and Molecular Biology, University of Nebraska Medical Center, Omaha, NE 68198-5870, USA; arpita.chatterjee@unmc.edu (A.C.); kliermann@unmc.edu (K.T.L.-W.); stakkar@unmc.edu (S.T.); becky.deegan@unmc.edu (R.E.O.-D.); 4 Department of Genetics, Cell Biology and Anatomy, University of Nebraska Medical Center, Omaha, NE 68198-5805, USA; nmutha@unmc.edu (N.M.);; 5 Nebraska Center for Integrated Biomolecular Communication, University of Nebraska-Lincoln, Lincoln, NE 68588-0304, USA

**Keywords:** transcriptomics, proteomics, metabolomics, bioinformatics, mouse bone marrow, radiation exposure

## Abstract

Large-scale radiation catastrophes and localized exposure incidents remain major threats to public health. Effective treatment following radiation exposure is challenging because radiation dose, timing, and affected tissues can vary widely between individuals. To better understand the biological response to radiation injury, we employed a multi-omics approach integrating transcriptomics, proteomics, and metabolomics. In this study, mouse bone marrow was analyzed at 1, 6, and 24 h after whole-body irradiation and compared with bone marrow from non-irradiated mice. Multivariate and univariate analyses revealed time-dependent changes across transcriptomic, proteomic, and metabolomic profiles. We further applied machine learning-based integrative analysis and biomarker discovery approaches to identify key molecular responses associated with radiation exposure. Integrated analysis showed that the acute radiation response in bone marrow included immune-related processes, followed by intermediate metabolic reprogramming, and later disruptions in cell cycle regulation. Biomarker analysis identified several promising candidates, including the transcripts *Sapcd2* and *Cd28*, proteins FRIL1 and CDK1, and the metabolites 3-hydroxyisobutyrate and taurine. Overall, our findings demonstrate that bone marrow from irradiated mice undergoes dynamic and coordinated molecular changes over time. These responses include activation of ferroptosis defense mechanisms, mitotic dysfunction, and pathways associated with programmed cell death.

## 1. Introduction

Radiation exposure is a considerable health risk for the general population. Ionizing radiation has the ability to penetrate through the body quickly and cause a wide array of consequences such as fertility issues, organ failure, cancer, and death [1]. Clinical assessment of radiation exposure requires medical personnel to diagnose the dosage, type, and timeline of an individual’s exposure to radiation before treatment [2]. These treatments are often complex, time-consuming, and require medical professionals with specific technical abilities [3]. However, early identification and medical intervention can drastically improve survival [4]. Therefore, investigation of the first 24 h after radiation exposure is necessary to aid medical professionals in early diagnosis and treatment.

Exposure to a radiation dose as low as 1 Gy puts a person at risk of illness and death, with an LD_50_ at 60 days of around 3.5 Gy if untreated [5]. The ensuing health effects from radiation exposure at high doses (>1 Gy) over short periods (<24 h) are known as acute radiation syndrome (ARS). ARS symptoms range from nausea, fatigue, and anemia that eventually lead to tissue damage, organ failure, and death [6]. Accordingly, ARS affects many parts of the human body, but it primarily targets the bone marrow, gastrointestinal system, cardiovascular system, and central nervous system at prolonged exposures [7]. Radiation-induced tissue damage appears to be dose-dependent, with bone marrow syndrome developing as low as 0.7 Gy, gastrointestinal syndrome developing above 6 Gy, and neurovascular syndrome occurring after 10+ Gy [4].

A biological explanation for the sensitivity of human tissues to radiation exposure is not completely understood. Tissues that contain rapidly dividing cells and stem cells are more prone to radiation-induced damage, suggesting that radiation damage critically interferes with cellular division and differentiation processes [8]. For example, radiation-induced damage to the bone marrow, also known as hematopoietic-ARS (H-ARS), results in the destruction of hematopoietic stem cells that reduces the ability of the body to produce new blood cells and platelets [9]. Our limited mechanistic understanding, especially within the first 24 h of radiation exposure, negatively impacts our ability to develop treatments and preventive measures for H-ARS [10,11]. Further, an effective treatment for H-ARS is dependent on an accurate and rapid diagnosis. Unfortunately, early detection is a challenge due to nuances of dose and radiation type, where limited diagnostic options are currently available. A multi-parameter approach based on symptoms, biophysical dosimetry, molecular biomarkers, and a more traditional approach of examining changes in hematology is commonly employed to appropriately evaluate patients during triage. Only a relatively small number of potential diagnostic biomarkers have been identified. For example, Acharya et al. found several serum microRNAs that were correlated with radiation dose [12]. Kim et al. and Yamaguchi et al. discovered protein modifications due to irradiation [13,14], while Shu et al. found that the inhibition of Caspase-1 mitigates post-irradiation hematopoietic stem cell damage [15]. Nevertheless, the dynamic cycle of gene expression after irradiation may make a single characterizing biomarker unreliable [16]. Instead, an array of chemical descriptors may be preferred to increase the accuracy of an H-ARS diagnosis [17].

Currently, there are nine FDA-approved therapeutics for the post-exposure treatment of H-ARS, which include genetically engineered growth factors and cytokines to facilitate the recovery of bone marrow cells [11]. There are relatively few options for the prevention of H-ARS. For example, some studies have shown that antioxidant supplementation may reduce the extent of radiation damage. Chen et al. observed that taurine supplementation supported bone growth and reduced oxidative stress [18], whereas Zhang et al. found that methionine promoted hematopoiesis after radiation exposure [19]. The tradeoff of using antioxidants as a prophylactic treatment of H-ARS has multiple limitations and concerns, including limited uptake and dietary restriction. Antioxidants are also poorly targeted, reduce endogenous reactive oxygen species (ROS) response, induce muscle fatigue, and reduce recovery from muscle use [20].

Individual molecules, metabolic or signaling pathways, or cellular processes that may prime the cell’s radiation response and improve its ability to remove ROS and repair DNA may lead to better treatments and outcomes for H-ARS, and for ARS in general. Transcriptomics, proteomics, and metabolomics (i.e., multi-omics) are valuable tools of systems biology that are routinely employed to understand a variety of molecular and cellular processes, to characterize mechanisms of action, and to identify disease biomarkers, among other applications [21,22,23]. A multi-omics approach is particularly advantageous for investigating radiation-induced damage to tissues like bone marrow because of the diversity of cellular processes impacted, as opposed to a single molecular target that is the typical subject of a study. Observing the overall changes in the transcriptome, proteome, and metabolome after radiation exposure may provide valuable insights into the underlying mechanisms of H-ARS while also identifying possible biomarkers for H-ARS diagnosis [24].

A temporal study is beneficial to study the longitudinal effects of radiation, survival mechanisms, and identify diagnostic biomarkers of ARS. Previous studies have investigated the temporal perturbations of ARS in the blood and serum of nonhuman primates. Vellichirammal et al. identified 20 transcriptional blood biomarkers characterizing the prophylactic treatment of BIO 300 in nonhuman primates 1 h and 4 h post-exposure to 5.8 Gy radiation [25]. The candidate biomarkers reflected molecular mechanisms of survival, including p53 regulation. In a separate study, Pannkuk et al. observed metabolic dysfunction in the serum of nonhuman primates between 24 h and 96 h after exposure to 7.2 Gy radiation [26]. Cheema et al. used logistic regression modeling to create a 4-analyte panel of biomarkers from a multi-omic (metabolomics, lipidomics, and proteomics) study that showed a 2- to 4-fold increase in serum abundance from 1 to 6 days after irradiation [27]. Collectively, these studies demonstrate the utility of molecular profiling for identifying radiation-associated biological perturbations and candidate biomarkers across multiple time scales. However, the complexity of the radiation response presents challenges for distinguishing early indicators of exposure from molecular changes associated with subsequent injury, compensatory responses, and recovery. Integrating complementary multi-omics measurements across multiple post-exposure time points may therefore provide a more comprehensive understanding of radiation-induced biological responses than any single analytical platform alone. Such an approach can also support the identification of time-dependent biomarkers and molecular pathways that may be relevant to the assessment of radiation exposure and the development of countermeasures for ARS.

In this study, we utilized a multi-omics approach to evaluate the molecular consequences of whole-body radiation on the bone marrow of mice. We observed changes in the transcriptome, proteome, and metabolome that correspond to the bone marrow cells undergoing oxidative stress, ferroptosis defense, glycolytic reprogramming, and mitotic catastrophe. We identified potential biomarkers of H-ARS and proposed a model of the temporal response of bone marrow cells 24 h post-radiation. These results highlight the effects of hematopoietic radiation exposure within the first 24 h to provide a foundation for future H-ARS mechanistic studies, as well as identify potential biomarkers for clinical assessments and therapeutic research.

## 2. Results

### 2.1. Transcriptomics

**Figure 1:**
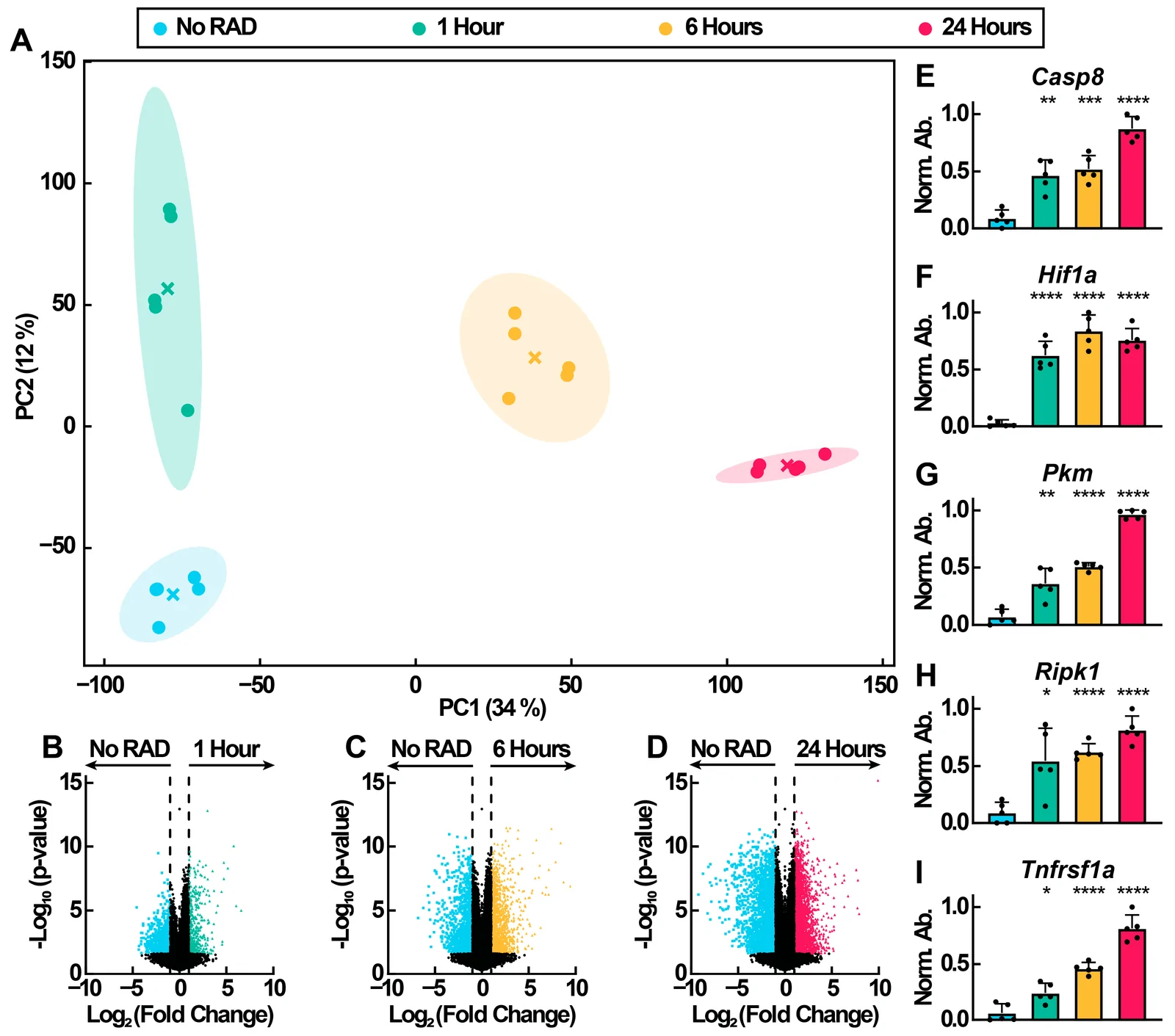
Overview of transcriptomics results. Colors used in all plots are by group: blue, No RAD; green, 1 h; yellow, 6 h; red, 24 h. (**A**) PCA scores plot for transcript changes in bone marrow during 1 h post-radiation, 6 h post-radiation, 24 h post-radiation, and No RAD. Ellipses indicate the group’s 95% confidence interval, with an X indicating the geometric center. (**B**) Volcano plot describing the pairwise comparison of 1 h vs. No RAD. (**C**) Volcano plot describing the pairwise comparison of 6 h vs. No RAD. (**D**) Volcano plot describing the pairwise comparison of 24 h vs. No RAD. For each volcano plot, the black points indicate transcripts with |Log_2_FC| < 1 and/or *p*_adj_ > 0.05. The colored points indicate transcripts with|Log_2_FC| > 1 and *p*_adj_ < 0.05. (**E**–**I**) Bar graphs of representative transcripts; *Casp8*, *Hif1a*, *Pkm*, *Ripk1*, and *Tnfrsf1a*. Bars are in order from left to right: No RAD, 1-hour, 6-hours, and 24-hours. Error bars of the bar graphs represent one standard deviation from the mean. Individual biological replicates overlay the bars as small black circles. The normalized data scale represents the Min-Max scaling of median-normalized data. The range of selected transcript-normalized abundances is formatted as [Min:Max]: (**E**) [9.11:19.5], (**F**) [7.73:20.81], (**G**) [76.56:230.81], (**H**) [9.78:23.65], and (**I**) [27.22:81.73]. Significance is denoted by an asterisk for comparison of each time point vs. the No RAD control. The significance was found using Student’s *t*-test, and a Benjamini–Hochberg FDR correction was applied. Significance is denoted in levels as follows: * *p*_adj_ < 0.05, ** *p*_adj_ < 0.01, *** *p*_adj_ < 0.001, **** *p*_adj_ < 0.0001.

Analysis of the transcriptomics dataset (Figure 1) revealed a total of 15,203 differentially expressed transcripts (*p*-value with Benjamini–Hochberg correction (*p*_adj_) ≤ 0.05) between the non-irradiated group (No RAD) and at least one of the irradiated groups at the 1, 6, or 24 h post-radiation time points. At 24 h post-irradiation, there were 5232 significantly altered transcripts (|Log_2_FC| > 1.0), with 2264 significantly upregulated and 2968 significantly downregulated transcripts. At the 24 h time point, the range of Log_2_FC values was from −9.2 to 7.9, with an average value for upregulated transcripts of Log_2_FC of 1.8 ± 0.8 and an average value for downregulated transcripts of Log_2_FC of −2 ± 1. Overall, 4795 transcripts were increased over the 24 h time course, 4229 were decreased, and 6179 exhibited a mixed response due to radiation exposure. A total of 1442 transcripts nearly recovered to, or rebounded beyond, pre-irradiation levels at the 24 h time point. A PCA scores plot demonstrated a clear separation between the post-radiation and No RAD groups, with the 24 h time point having the greatest separation from No RAD (Figure 1A). The temporal response over the course of the 24 h may be reflected in the two-dimensional positional change in group scores in the PCA. The temporal nature of the radiation response may also be defined by an increase in the number of significantly increasing transcripts at each successive time point, shown in the volcano plots in Figure 1B–D.

The maximum Log_2_FC transcript at 24 h was *Plb1* (Log_2_FC = 7.9, *p*_adj_ < 0.001). The minimum Log_2_FC transcript at 24 h was *Rag1* (Log_2_FC = −9.2, *p*_adj_ < 0.0001). When examining the significantly changing transcripts, we noticed major changes for a number of transcripts that encode proteins important for cell cycle, cell stress management, and programmed cell death. For example, the 24 h post-radiation time point had decreased abundance of *Mcm2* (Log_2_FC = −1.3, *p*_adj_ < 0.0001), *Cdk1* (Log_2_FC = −1.0, *p*_adj_ < 0.0001), and transcription factor *E2f4* (Log_2_FC = −2.1, *p*_adj_ < 0.0001). The cell cycle driver *Cdk1* showed an increase in abundance at 1 h post-radiation, followed by a progressive decline through to the 24 h post-radiation time point. The 24 h post-radiation time point had increased abundance of p53 target *Phlda3* (Log_2_FC = 5.6, *p*_adj_ < 0.0001), p21-encoding transcript *Cdkn1a* (Log_2_FC = 4.8, *p*_adj_ < 0.0001), the pro-apoptotic transcripts *Bax* (Log_2_FC = 1.7, *p*_adj_ < 0.0001) and *Casp8* (Log_2_FC = 0.9, *p*_adj_ < 0.0001) (Figure 1E), and the hypoxia regulating transcript *Hif1a* (Log_2_FC = 1.1, *p*_adj_ < 0.0001) (Figure 1F). The transcript for the glycolytic enzyme *Pkm* (Log_2_FC = 1.4, *p*_adj_ < 0.0001) (Figure 1G) displayed a time-dependent increase in normalized transcript abundance across the 1, 6, and 24 h post-radiation time points relative to the No RAD group [28,29]. The necroptosis transcript *Ripk1* (Log_2_FC = 0.9, *p*_adj_ < 0.0001) and *Tnfrsf1a* (Log_2_FC = 1.2, *p*_adj_ < 0.0001) increased (Figure 1H,I). Furthermore, the glutathione-regulating enzyme *Gsr* (glutathione reductase; Log_2_FC = 1.4, *p*_adj_ < 0.0001) showed a progressive transcriptional upregulation starting at the 1 h time point and peaking at 24 h post-radiation [30]. See the Supporting Information (Tables S1–S3) for a complete list of dysregulated transcripts including time-dependent fold changes and FDR-corrected *p*-values.

### 2.2. Proteomics

**Figure 2:**
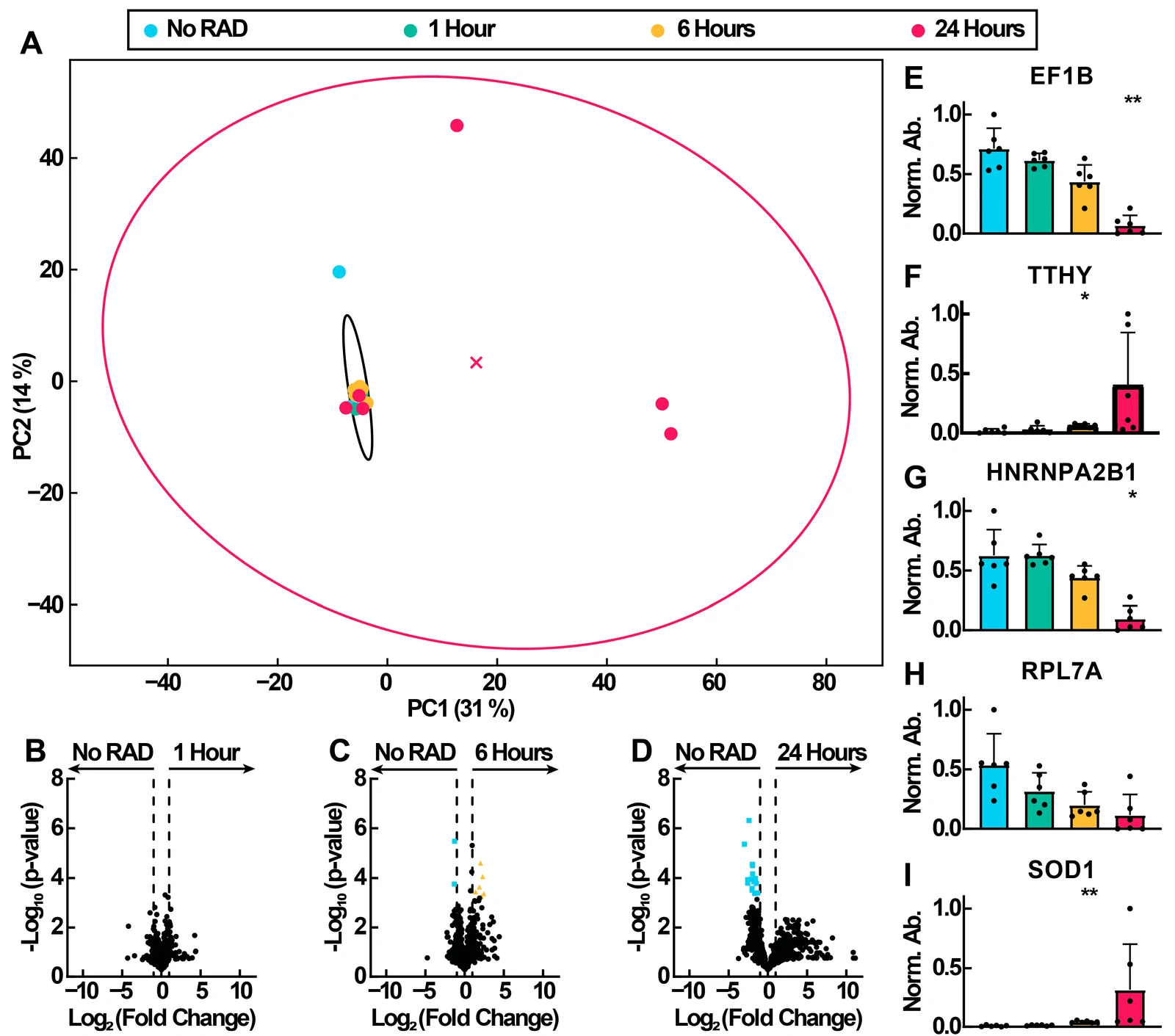
Overview of proteomics results. Colors used in all plots are by group: blue, No RAD; green, 1 h; yellow, 6 h; red, 24 h. (**A**) PCA scores plot for proteomic changes in bone marrow during 1 h post-radiation, 6 h post-radiation, 24 h post-radiation, and No RAD. All replicates are designated by group color. The 24 h group 95% confidence ellipse is denoted in the appropriate color, and all other groups have a combined 95% confidence ellipse denoted in black. The geometric center of the 95% confidence ellipse is indicated by an X. (**B**) Volcano plot describing the pairwise comparison of 1 h vs. No RAD. (**C**) Volcano plot describing the pairwise comparison of 6 h vs. No RAD. (**D**) Volcano plot describing the pairwise comparison of 24 h vs. No RAD. For each volcano plot, the black points indicate proteins with |Log_2_FC| < 1 and/or *p*_adj_ > 0.05. The colored points indicate proteins with |Log_2_FC| > 1 and *p*_adj_ < 0.05. (**E**–**I**) Bar graphs of representative proteins: EF1B, TTHY, HNRNPA2B1, RPL7A, and SOD1. Bars are in order from left to right: No RAD, 1-hour, 6-hours, and 24-hours. Error bars of the bar graphs represent one standard deviation from the mean. Individual biological replicates overlay the bars as small black circles. The normalized data scale represents the Min-Max scaling of median-normalized data. The range of selected protein normalized abundances is formatted as [Min:Max]: (**E**) [0.08:3.62], (**F**) [0.08:5.99], (**G**) [0.10:11.15], (**H**) [0.19:11.48], and (**I**) [9.82:333.23]. Significance is denoted by an asterisk for comparison of each time point vs. the No RAD control. The significance was found using Student’s *t*-test, and a Benjamini–Hochberg FDR correction was applied. Significance is denoted in levels as follows: * *p*_adj_ < 0.05, ** *p*_adj_ < 0.01.

Unlike the transcriptomics data, the proteomics PCA scores plot captures little time-dependent variation (Figure 2A). Nevertheless, protein-level analyses revealed modest remodeling of the proteome over the 24 h study period. Untargeted proteomic analysis of bone marrow from irradiated mice yielded a total identification of 795 proteins. Of the 795 identified, 27 proteins were significantly different in at least one post-radiation time point after pairwise comparison with No RAD (*p*_adj_ < 0.05). Of the significant proteins, nine increased over the time course, 14 decreased, and four exhibited a mixed response due to radiation exposure. Only one protein (26S proteasome regulatory subunit 4 (PRS4)) nearly recovered to, or rebounded beyond, pre-irradiation levels by the end of the 24 h time period. At the 24 h time point, the Log_2_FC values for significant (*p*_adj_ < 0.05) proteins ranged from −3.0 to −1.3, with the decreased proteins possessing an average Log_2_FC of −2.0 ± 0.5. The time-dependent variation in the bone marrow proteome is more evident in the individual protein abundance changes summarized in the volcano plots (Figure 2B–D).

While most of the proteome changes occurred at 24 h, notable changes were also observed at other time points. The maximum Log_2_FC protein at 24 h was the large ribosomal subunit protein eL36 (RL36) (Log_2_FC = 11.2, *p*_adj_ *=* 0.3). The minimum Log_2_FC significant protein at 24 h was elongation factor 1-beta (EF1B) (Log_2_FC = −3.0, *p*_adj_ < 0.01) (Figure 2E). At the 1 h time point, the ferritin light chain 1 (FRIL1) (1 h, Log_2_FC = 1.1, *p*_adj_ *=* 0.1) was identified as one of the most increased proteins compared to No RAD (Figure S2). FRIL1 is the primary cellular iron storage protein. A transporter protein critical for hormone signaling, transthyretin (TTHY), was found to increase significantly at 6 h (Log_2_FC = 1.4, *p*_adj_ < 0.01) (Figure 2F). Interestingly, two nuclear functional proteins exhibited opposite trends. Lamin B1 (LMNB1) (Log_2_FC = 3.5, *p*_adj_ = 0.1) increased at the 6 h post-radiation time point, while the heterogeneous nuclear ribonucleoproteins A2/B1 (HNRNPA2B1) (Log_2_FC = −2.6, *p*_adj_ < 0.05) significantly decreased at 24 h (Figure 2G). Another protein that decreased at 24 h was the large ribosomal protein 7a (RPL7A) (Log_2_FC = −2.1, *p*_adj_ = 0.1) (Figure 2H). The antioxidant protein superoxide dismutase 1 [Cu/Zn] (SOD1) (Log_2_FC = 1.0, *p*_adj_ < 0.01) significantly increased at the 6 h post-radiation time point (Figure 2I). See the Supporting Information (Tables S9–S11) for a complete list of dysregulated proteins including time-dependent fold changes and FDR-corrected *p*-values.

### 2.3. Metabolomics

**Figure 3:**
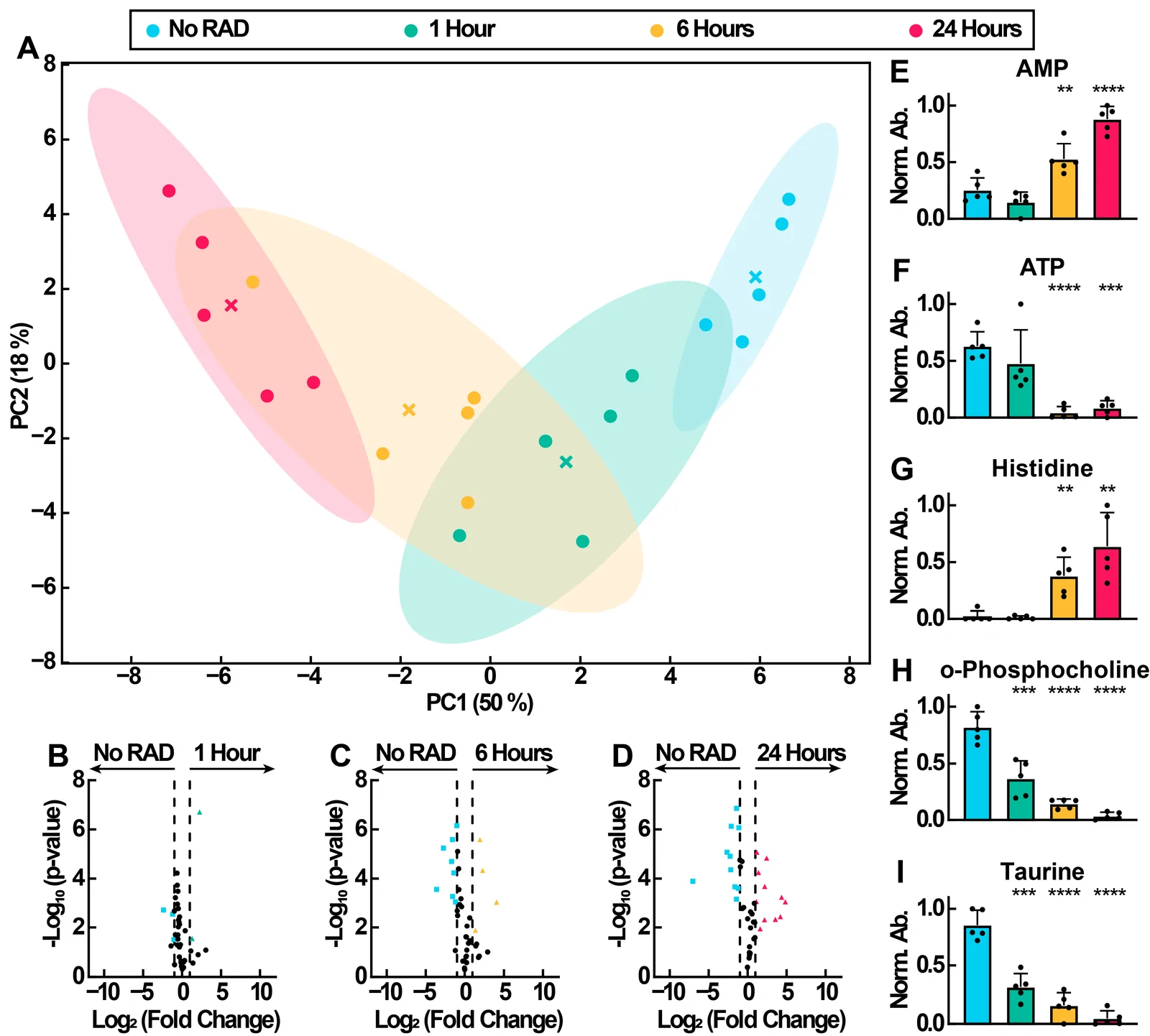
Overview of metabolomics results. Colors used in all plots are by group: blue, No RAD; green, 1 h; yellow, 6 h; red, 24 h. (**A**) PCA scores plot for metabolomic changes in bone marrow during 1 h post-radiation, 6 h post-radiation, 24 h post-radiation, and No RAD. All replicates are designated by group color. Ellipses indicate the group 95% confidence interval, with an X indicating the geometric center. (**B**) Volcano plot describing the pairwise comparison of 1 h vs. No RAD. (**C**) Volcano plot describing the pairwise comparison of 6 h vs. No RAD. (**D**) Volcano plot describing the pairwise comparison of 24 h vs. No RAD. For each volcano plot, the black points indicate metabolites with |Log_2_FC| < 1 and/or *p*_adj_ > 0.05. The colored points indicate metabolites with |Log_2_FC| > 1 and *p*_adj_ < 0.05. (**E**–**I**) Bar graphs of representative metabolites: adenosine monophosphate (AMP), adenosine triphosphate (ATP), histidine, o-phosphocholine, and taurine. Bars are in order from left to right: No RAD, 1-hour, 6-hours, and 24-hours. Error bars of the bar graphs represent one standard deviation from the mean. Individual biological replicates overlay the bars as small black circles. The normalized data scale represents the Min-Max scaling of median-normalized data. The range of selected metabolite normalized abundances is formatted as [Min:Max]: (**E**) [0.86:4.38], (**F**) [0.21:3.69], (**G**) [0.15:0.71], (**H**) [1:6.07], and (**I**) [13.66:35.09]. Significance is denoted by an asterisk for comparison of each time point vs. the No RAD control. The significance was found using Student’s *t*-test, and a Benjamini–Hochberg FDR correction was applied. Significance is denoted in levels as follows: ** *p*_adj_ < 0.01, *** *p*_adj_ < 0.001, **** *p*_adj_ < 0.0001.

Untargeted metabolomic profiling of bone marrow from irradiated mice identified 72 metabolites by NMR, of which 40 exhibited significant abundance changes at one or more post-irradiation time points relative to No RAD controls (*p*_adj_ < 0.05). Across the 24 h study period, 16 metabolites increased, 14 decreased, and 10 displayed mixed temporal responses to radiation exposure. Notably, glutathione was the only metabolite to recover to, or exceed, pre-irradiation levels by 24 h (*p*_adj_ < 0.05). By 24 h post-irradiation, metabolite fold changes spanned a broad range (Log_2_FC from −7.0 to 4.8). Increased abundance metabolites exhibited an average Log_2_FC of 3 ± 1, whereas decreased abundance metabolites exhibited an average Log_2_FC of −2 ± 2. Collectively, these findings suggest a progressive metabolic response that intensified over time following radiation exposure. This temporal trajectory was readily apparent in the PCA scores plot (Figure 3A), where samples became increasingly separated from No RAD controls with increasing time after irradiation. Pairwise comparisons further resolved these shifts, as illustrated by the volcano plots in Figure 3B–D. Consistent with the PCA results, the number of significantly altered metabolites (|Log_2_FC| > 1, *p*_adj_ < 0.05) increased from only four at 1 h post-irradiation to twenty at 24 h, highlighting the expanding metabolic perturbation over time.

Separation of groups in PCA suggests that the energy state of bone marrow cells was the most perturbed at 24 h. At 24 h, the energy metabolites ATP (Log_2_FC = −2.3, *p*_adj_ < 0.001), ADP (Log_2_FC = −1.2, *p*_adj_ < 0.01), and AMP (Log_2_FC = 1.2, *p*_adj_ < 0.0001) were found to be disrupted (Figure 3E,F). Histidine, a metal-chelating amino acid, increased (Log_2_FC = 4.8, *p*_adj_ < 0.01) over the 24 h period (Figure 3G). o-Phosphocholine, the polar head group of many membrane lipids, decreased (Log_2_FC = −2.1, *p*_adj_ < 0.0001) over the 24 h period (Figure 3H). Antioxidant metabolite taurine (Log_2_FC = −1.1, *p*_adj_ < 0.0001) was also found to be perturbed at all post-radiation time points (Figure 3I). See the Supporting Information (Tables S18–S20) for a complete list of dysregulated metabolites including time-dependent fold changes and FDR-corrected *p*-values.

### 2.4. Integrated Analysis

Pathway analysis of the different datasets, including the significantly differentially expressed transcripts, proteins, and metabolites, was performed to investigate correlations between -omics analyses to further understand biological impact. From a KEGG pathway analysis, the top six enriched transcriptomic pathways were (1) ribosome (156 transcripts, *p* < 0.0001), (2) cell cycle (131 transcripts, *p* < 0.0001), (3) cellular senescence (121 transcripts, *p* < 0.0001), (4) HIF-1 signaling pathway (79 transcripts, *p* < 0.0001), (5) apoptosis (96 transcripts, *p* < 0.0001), (6) p53 signaling pathway (58 transcripts, *p* < 0.0001). The top five enriched proteomics (using proteins with *p* < 0.05) pathways were (1) regulation of PD-L1 (CD274) transcription (28/41 proteins, *p* < 0.0001), (2) epigenetic regulation of adipogenesis genes by MLL3 and MLL4 complexes (26/45 proteins, *p* < 0.0001), (3) MLL4 and MLL3 complexes regulation of expression of PPARG target genes in adipogenesis and hepatic steatosis (26/45 proteins, *p* < 0.0001), (4) inhibition of DNA recombination at telomere (24/43 proteins, *p* < 0.0001), (5) PRC2 methylates histones and DNA (28/51 proteins, *p* < 0.0001). The top five enriched metabolomic pathways were (1) alanine, aspartate, and glutamate metabolism (6/28 metabolites, *p* < 0.01), (2) glyoxylate and dicarboxylate metabolism (6/32 metabolites, *p* = 0.01), (3) arginine biosynthesis (4/14 metabolites, *p* = 0.01), (4) one carbon pool by folate (5/26 metabolites, *p* = 0.01), (5) histidine metabolism (4/16 metabolites, *p* = 0.01). See the Supporting Information (Tables S7, S15 and S24) for a complete list of dysregulated pathways including the list of associated transcripts, proteins and metabolites.

Joint pathway enrichment analysis of the significant transcripts and proteins revealed a temporal radiation response identified by g:Profiler. The analysis is dominated by transcriptomic data since there are more statistically significant transcripts altered by radiation than significant proteins or metabolites. At 1 h post-irradiation, the acute response was characterized by the downregulation of immune-related processes, namely, B-cell mediated immunity, antigen-binding, and adaptive immune response. By 6 h post-irradiation and persisting through the 24 h time point, there was a consistent downregulation of regulators of transcription and cell-cycle progression (i.e., *E2f1*, *Atf4*, *Cdk2*, and YBX1). A full list of joint pathways is listed in Supporting Information (Table S26).

In total, 742 of the 795 proteins detected also had an associated transcript detected. Of the 742 proteins, 199 had a strong correlation (Pearson correlation > 0.8) with their associated transcript based on Log_2_FC over the time course, while 130 were strongly anti-correlated (Pearson correlation < −0.8). These anti-correlated proteins were denoted as discordant. Discordance in the changes of six key transcripts and proteins was observed. Here we report the Log_2_FC of the transcripts and proteins at 24 h post-radiation exposure as well as the temporal Pearson correlation. *Hk1*, a regulator of glycolysis, experienced downregulated transcript levels at the 24 h time point (Log_2_FC = −0.2, *p*_adj_ < 0.01), while the associated protein HK1 increased (Log_2_FC = 5.0, *p*_adj_ = 0.2) (Pearson correlation = −0.9828). *Gpx4*, a transcript involved in the ferroptosis pathway and associated with the regulation of glutathione-dependent peroxide reduction, experienced downregulated transcript levels (Log_2_FC = −0.5, *p*_adj_ < 0.0001) and upregulation of the associated protein GPX4 (Log_2_FC = 3.3, *p*_adj_ = 0.3) (Pearson correlation = −0.9996). Transcripts for key DNA regulators *Lmnb1* (Log_2_FC = −0.6, *p*_adj_ < 0.0001) and *Rcc2* (Log_2_FC = −0.8, *p*_adj_ < 0.0001) were downregulated, while their corresponding protein levels were upregulated: LMNB1 (Log_2_FC = 6.2, *p*_adj_ = 0.3) (Pearson correlation = −0.9972) and RCC2 (Log_2_FC = 8.2, *p*_adj_ = 0.2) (Pearson correlation = −0.8219). There was also discordance between transcription and protein synthesis regulator transcripts *Smc2* (Log_2_FC = −1.2, *p*_adj_ < 0.0001) (Pearson correlation = −0.9601) and *Eif3j2* (Log_2_FC = −1.1, *p*_adj_ < 0.05) (Pearson correlation = −0.9879) and the associated proteins SMC2 (Log_2_FC = 4.2, *p*_adj_ = 0.3) and EIF3J2 (Log_2_FC = 2.8, *p*_adj_ = 0.3). A full list of Pearson correlations can be found in Table S27.

### 2.5. Potential H-ARS Biomarker Determination

**Figure 4:**
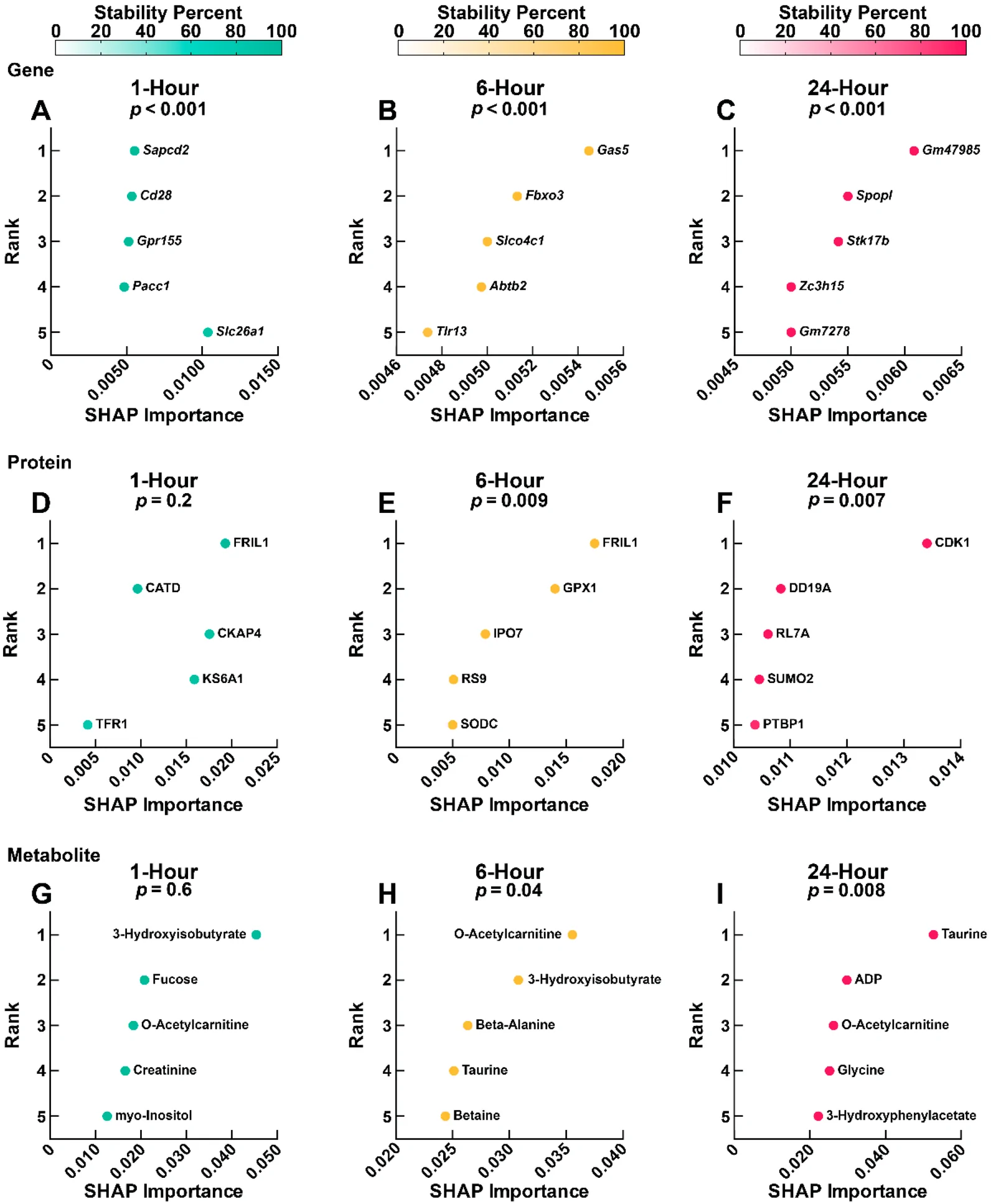
Overview of biomarker analysis displaying the top **5** compounds of interest at each time point for each -omics analysis. Bubble graphs are colored based on the stability percent metric of the LOOCV. The *p*-value for each Random Forest model was calculated by permutation testing with 1000 iterations. Transcriptomic analysis is displayed in the top row with (**A**) 1 h, (**B**) 6 h, and (**C**) 24 h analyses compared with No RAD. Proteomic analysis is displayed in the second row with (**D**) 1 h, (**E**) 6 h, and (**F**) 24 h analyses compared with No RAD. Metabolomic analysis is displayed in the third row with (**G**) 1 h, (**H**) 6 h, and (**I**) 24 h analyses compared with No RAD.

Complementing the classical enrichment analysis, a machine learning approach identified biomarker signatures that distinguish irradiated vs. No RAD control samples. Random forest models (*n* = 10 or 12 total samples, representing 5 or 6 biological replicates per group) were trained using Leave-One-Out Cross-Validation (LOOCV) combined with permutation testing. At 1 h post-radiation, transcriptomic models demonstrated high predictive accuracy (LOOCV accuracy 100%, permutation *p* ≤ 0.001), driven by transcripts such as *Sapcd2* (Shapley Additive exPlanations (SHAP) = 0.006, 100% fold stability). In contrast, 1 h proteomic and metabolomic models did not achieve predictive significance. However, at later time points, predictive accuracy improved substantially. At both 6 h and 24 h, all models achieved ≥ 90% accuracy (*p* < 0.05). By 24 h, highly stable biomarkers were found across all three -omics layers, including the mitotic driver CDK1 (SHAP = 0.013, 100% fold stability), the iron storage protein FRIL1 (SHAP = 0.011, ≥83% [10/12] fold stability), and the antioxidant metabolite taurine (SHAP = 0.053, 100% fold stability) (Figure 4). A full list of SHAP scores is listed in Supporting Information (Tables S8, S16 and S25).

## 3. Discussion

### 3.1. Summary

**Figure 5:**
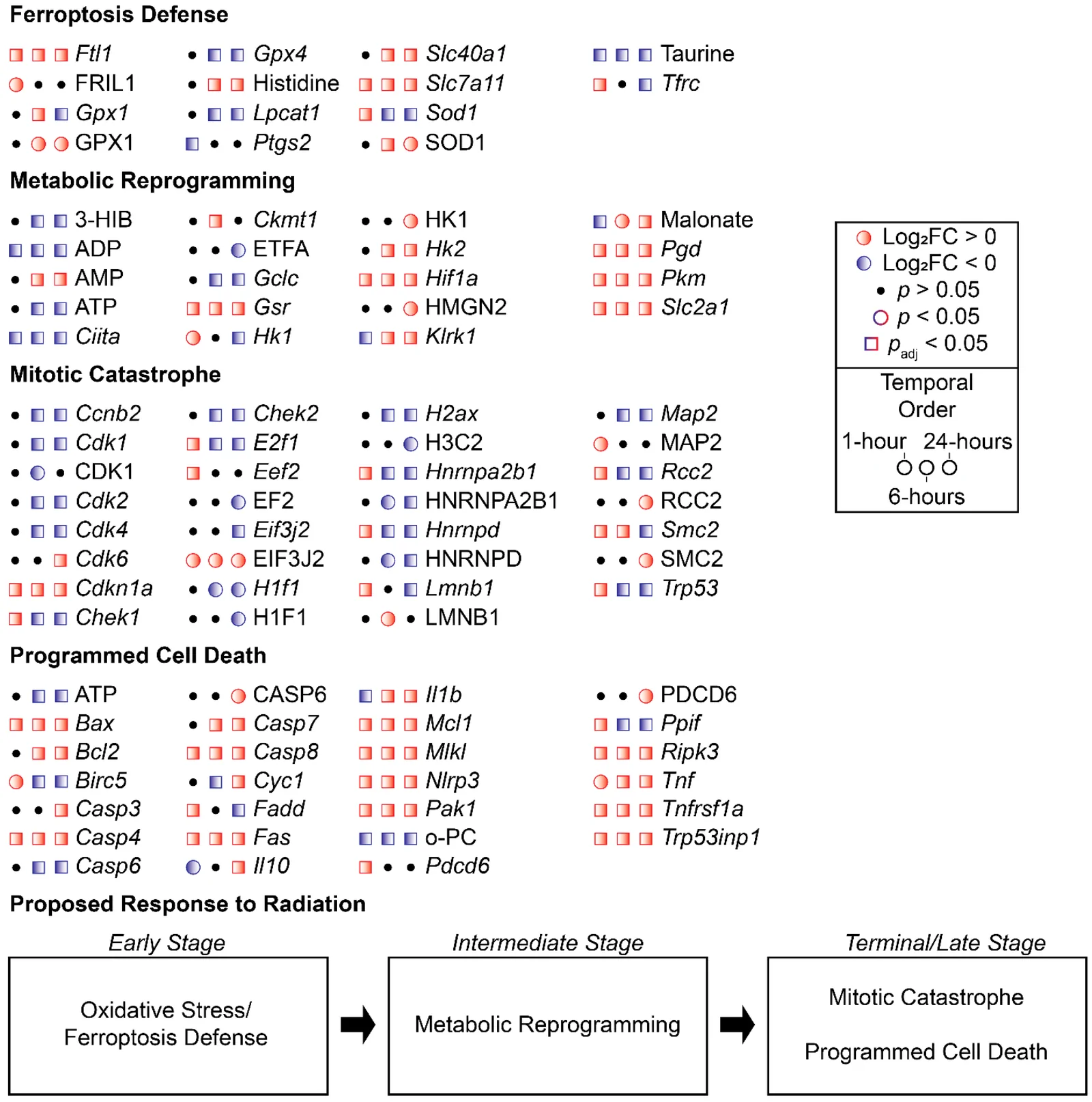
Overview of discussion topics: ferroptosis defense, metabolic reprogramming, mitotic catastrophe, programmed cell death, and how each topic fits into the general radiation timeline. Circles or squares next to each compound of interest are colored based on significance and fold change and ordered temporally. Red (gradient right) indicates an increase (Log_2_FC > 0), and blue (gradient left) indicates a decrease (Log_2_FC < 0). Black dots represent comparisons with *p* > 0.05, circles represent comparisons with *p* < 0.05, and squares indicate comparisons with *p*_adj_ < 0.05. Non-standard abbreviations are 3-HIB for 3-hydroxyisobutyrate, o-PC for o-phosphocholine. All other abbreviations are transcript/protein abbreviations or adenosine phosphate abbreviations (ADP, AMP, and ATP).

Furthering our understanding of radiation-induced cell death in H-ARS may provide valuable insights for developing novel radiation countermeasures and effective diagnostic tools. Towards this end, we used a multi-omics and multi-platform approach to investigate the molecular mechanisms and biomarkers associated with H-ARS in an animal model. This combined transcriptomics, proteomics, metabolomics, and pathway analysis of bone marrow cells extracted from mice following whole-body radiation exposure (5.96 Gy) revealed a distinct response to radiation. Figure 5 summarizes four of the biological processes observed to be changing in bone marrow cells following radiation exposure: (1) ferroptosis defense, (2) metabolic reprogramming, (3) mitotic catastrophe, and (4) programmed cell death. It is important to note that while the volcano plots in Figure 1, Figure 2 and Figure 3 display a predefined significance criterion (*p*_adj_ < 0.05 and |Log_2_FC| > 1), Figure 5 includes molecules with Log_2_FC > 0 and Log_2_FC < 0 to reflect all biologically relevant expression changes during the four observed biological processes. In Figure 5, we also include compounds meeting either the raw *p*-value threshold (*p* < 0.05) or Benjamini–Hochberg adjusted *p*-value threshold (*p*_adj_ < 0.05) to reflect the full range of statistical evidence. The rationale for reporting both FDR-corrected and non-FDR-corrected significant results is to provide transparency and a more complete representation of the data while acknowledging the balance between reducing Type I errors achieved through multiple comparison correction and the increased risk of Type II errors that may exclude potentially meaningful biological results [31,32]. Furthermore, a machine learning analysis of the multi-omics data set identified a highly stable panel of 180 top predictive biomarker signatures for H-ARS across all three -omics layers. A list of the top 20 biomarkers for each -omics can be found in the Supporting Information (Tables S8, S16 and S25). A detailed discussion and description of the bone marrow biomolecules identified to be impacted by an exposure to radiation follows.

### 3.2. Early Oxidative Stress and Ferroptosis-Defense Response

Antioxidant defense was evidenced by the increased abundance of antioxidant proteins GPX1 and SOD1. Our proteomics analysis revealed an increase in the abundance of FRIL1 at the 1 h time point following radiation treatment. FRIL1 is a subunit of ferritin that captures free iron to inhibit its interaction with radiation-induced hydrogen peroxide and prevents the Fenton reaction. The Fenton reaction is a biological oxidation process that occurs when Fe(II) and H_2_O_2_ produce hydroxyl radicals within the body. The increased abundance of FRIL1 may therefore contribute to limiting lipid peroxide accumulation and reduce the susceptibility to ferroptosis [33]. The associated transcript *Ftl1* was increased over each time period. FRIL1 abundance supports iron sequestration as an early response to radiation-induced damage to counteract the threat of ferroptosis and the ensuing oxidative stress. The transcript that encodes the transferrin receptor protein 1, *Tfrc*, also increased at 1 h, but was decreased at 24 h. Bar graphs of abundances over time for all transcripts, proteins, and metabolites listed in this section can be found in Figure S2.

In addition to the proteins outlined above, metabolomics analysis revealed a temporal increase in histidine at 6 h and 24 h post-radiation, and a temporal decrease in taurine at 1 h, 6 h, and 24 h post-radiation. The final steps of the ferroptosis pathway involve lipid peroxidation before cell death, which is driven by the Fenton reaction. Histidine is an antioxidant and reactive oxygen species scavenger that can limit the Fenton reaction by scavenging and chelating metal ions to limit cell damage [34]. The increase in histidine abundance at 6 h and 24 h post-radiation may contribute to mitigating oxidative stress associated with ferroptosis through iron chelation and limiting hydroxyl radical formation. Indeed, a correlation between radiation resistance and histidine concentration has been previously reported [35]. The dynamic decrease in taurine may also be associated with changes in the cellular response to oxidative stress [18].

### 3.3. Metabolic Reprogramming

An increase in malonate and a perturbation in ATP/ADP/AMP ratios provide evidence for a metabolic shift. Increased levels of cellular malonate induce aerobic glycolysis by inhibiting succinate dehydrogenase, a key enzyme for the TCA cycle and the electron transport chain [36]. These changes may be associated with reduced mitochondrial oxidative metabolism and a relative increase in glycolytic ATP production. Similarly, high levels of cellular AMP typically activate glycolysis by binding to phosphofructokinase; however, AMP is also involved in the generation of ATP through the oxidative phosphorylation pathway by binding AMP-activated protein kinase during periods of high energy demand [37]. Transcriptomics also identified an increase in *Ckmt1* (6 h, Log_2_FC = 7.1, *p*_adj_ < 0.0001) that may corroborate the ATP shortage. Therefore, the increased abundance of malonate and AMP may suggest changes in cellular energy metabolism as part of the cellular radiation response. Bar graphs of abundances over time for all transcripts, proteins, and metabolites listed in this section can be found in Figure S3.

Enrichment analysis also shows a transition toward “immune system processes” and “defense response” (Table S26). A significant upregulation of the transcripts of glycolytic enzymes *Hk2* and *Pkm* was observed. The HK2 protein was not identified, but the HK1 protein that catalyzes the initial step of glycolysis increased at 24 h. The PKM protein was identified, but was not significant at any time point compared to No RAD. The cell may be foregoing oxidative phosphorylation for glycolysis, suggestive of Warburg-like metabolic reprogramming [38,39]. An increase in *Hif1a* was observed at 1 h, 6 h, and 24 h post-radiation. This increase suggests a disruption in glucose metabolism, potentially indicating the Warburg effect [40,41].

### 3.4. Cell-Cycle Suppression, Transcript–Protein Discordance, and Mitotic Catastrophe

Proteomics analysis shows an increase in the abundance of mitotic drivers at 24 h post-radiation relative to No RAD, specifically for RCC2 and SMC2. However, the transcriptomics data showed downregulation of *Rcc2* and *Smc2* mRNA. The discordance between the increased abundances of these mitotic proteins and decreased *Rcc2* and *Smc2* transcript levels may reflect altered regulation of mitotic proteins that may indicate limited mitotic progression or mitotic catastrophe [42]. For example, increased abundances at 24 h of cyclin G1: *Ccng1* (Log_2_FC = 3.5, *p*_adj_ < 0.0001), cyclin dependent kinase inhibitor 1A: *Cdkn1a* (Log_2_FC = 4.8, *p*_adj_ < 0.0001), and MDM2 proto-oncogene: *Mdm2* (Log_2_FC = 1.4, *p*_adj_ < 0.0001) suggest alterations in p53-regulated inhibition of mitosis, which could limit the propagation of damaged DNA [43]. Other mitotic driver transcripts involved in mitosis regulation that are downregulated are *Cdk1*, *Ccnb2*, and *E2f1* [44,45]. The GTPase protein EF2, which is important for translation, also had decreased abundance at 24 h. Bar graphs of abundances over time for all transcripts and proteins listed in this section can be found in Figure S4.

Other observations consistent with mitotic catastrophe include depletion of histones H1-1 (H1F1) and H3C2 24 h post-radiation. The decrease in histone abundance is consistent with the chromatin destabilization and transcriptional failure that is often seen in radiation damage [46]. This decrease in histone abundance is also consistent with the transcriptomics findings for *H2ax* and *H1f1* but not *H3c2*. Finally, a decrease in the *Lmnb1* transcript was observed at 24 h post-radiation, while the associated protein significantly increased at 6 h and was unchanged at 24 h post-radiation. A decrease in *Lmnb1* has been shown to indicate cell cycle arrest [47]. LMNB1 is crucial for cellular localization of DNA in the nucleus, nuclear lamina dynamics during mitosis, and chromatin organization and an increase in the LMNB1 protein is associated with mitotic catastrophe and cell senescence [48]. The discordance between transcript and protein abundances may corroborate the previous statement about a buildup of mitotic proteins. However, LMNB1’s role in mitotic catastrophe still needs to be explored. Collectively, the transcriptomic and proteomic findings at 24 h post-radiation suggest dysregulated mitotic progression, chromatin destabilization, and cell cycle arrest, which are consistent with mitotic catastrophe.

### 3.5. Programmed Cell Death

Our multi-omics results may indicate that bone marrow cells are undergoing two mechanisms of programmed cell death: apoptosis and necroptosis [49]. Apoptosis is a gene-regulated and caspase-dependent form of programmed cell death, while necroptosis is a non-apoptotic, caspase-independent pathway of cell death that causes cell swelling, membrane rupture, and inflammation [50]. A change in the expression of key apoptotic genes *Bax*, *Cyc1*, *Mcl1*, *Casp3*, *Casp7*, *Casp8* and the CASP6 protein was observed post-radiation. An increase in key necroptosis transcripts such as *Tnfrsf1a*, *Ripk3*, and *Mlkl* was detected within 24 h post-radiation. The necroptotic transcript, *Ppif*, increased at 1 h and then decreased. A decrease in the abundance of metabolites ATP and o-phosphocholine was detected within 24 h post-radiation. Bar graphs of abundances over time for all transcripts, proteins, and metabolites listed in this section can be found in Figure S5.

Apoptosis occurs in response to specific signals from two main cellular processes, the intrinsic and extrinsic pathways [51]. The intrinsic apoptosis pathway is tightly regulated and is initiated by internal signals such as DNA damage [52]. This triggers both pro-apoptotic and anti-apoptotic members of the Bcl protein family to activate, such as *Bcl2*, *Mcl1*, and *Bax*. Dysregulation of the pro- and anti-apoptotic protein ratios, as well as post-translational protein modifications, modulates pathway activity. Transcriptomic analysis identified a significant increase in *Mcl1* and *Bax* from 1 h through 24 h and *Bcl2* from 6 h through 24 h. *Bcl2* and *Mcl1* are anti-apoptotic regulators, while *Bax* is a pro-apoptotic regulator. An increase in *Bax* is a classic indicator of apoptosis; however, an increase in the anti-apoptotic transcripts *Bcl2* and *Mcl1* is typically associated with apoptotic protection. This apparent contradiction may suggest an attempt by the cell to protect itself from stress [53]. Alternatively, post-translational modifications of BCL-2 may result in dysregulation in the apoptotic pathway; therefore, the increase in *Bcl2* does not strictly indicate halting of the apoptotic process. There was upregulation of the pyroptotic transcript *Casp4* levels at all time points post-radiation and a decrease in the apoptotic transcripts *Casp6* and *Cyc1* abundance at 6 h post-radiation. Conversely, the *Casp6* transcript levels were discordant with the proteomics data, which showed an increase in CASP6 at 24 h post-radiation.

The extrinsic apoptosis pathway is triggered by external molecular signals that bind to membrane-bound receptors, such as tumor necrosis factor (TNF) receptors. Ligand binding to the TNF receptor activates the FADD protein to be recruited to the receptor complex, which causes CASP8 activation followed by downstream activation of effector caspases-3, 6, and 7 to execute apoptosis. In necroptosis, activation of TNF receptors causes recruitment of the RIPK1 and RIPK3 proteins. RIPK1 and RIPK3 autophosphorylate and transphosphorylate to form a necrosome to subsequently activate MLKL, which leads to plasma membrane rupture [54]. An increase in *Casp8* transcripts throughout all post-radiation time points, as well as a decrease in *Fadd* transcripts at 24 h post-radiation, was observed. The programmed cell death 6 protein (PDCD6), which is involved in the signaling in extrinsic apoptosis, was increased at 24 h. These findings may suggest a change in programmed cell death [55]. FAS was not significantly changed at any hour, but its associated transcript significantly increased at all post-radiation time points. Finally, an increase in the expression of key genes involved in necroptosis was also observed. *Tnf*, *Tnfrsf1a*, and *Casp8* are involved in both extrinsic apoptosis and necroptosis pathways, which suggests multiple cell death pathways may be activated after radiation exposure.

*Tnf*, *Tnfrsf1a*, *Ripk3* and *Mlkl* were upregulated at 24 h post-radiation. The statistically significant changes in the expression of necroptosis pathway genes provide important insight on the state of bone marrow cells post-radiation and suggests necroptotic cell death; however, it is important to note that the associated proteins were not detected in the proteomics analysis of the bone marrow cells. Metabolomics analysis revealed a decrease in abundance at 24 h post-radiation of related metabolites. The observed depletion of ATP is a hallmark of necroptosis since ATP is consumed for necrosome formation [50]. o-Phosphocholine is involved in a key step in the synthesis of phosphatidylcholine, which is the major component of plasma membranes. A dysregulation in o-phosphocholine levels may suggest compromised cell membrane integrity [56,57]. Overall, our multi-omics analysis suggests the activation of both apoptosis and necroptosis pathways through dysregulation of key pathway transcripts, proteins, and downstream metabolites.

### 3.6. Candidate Biomarkers

The multi-omics analysis combined with random forest models, LOOCV, and 1000-iteration permutation testing identified a highly stable panel of candidate biomarkers associated with H-ARS. The application of machine learning analysis to our in-depth multi-omics study strengthened our understanding of H-ARS by identifying the most biologically relevant biomarkers with an expected greater predictive power and accuracy compared to traditional single -omics strategies. Random forest models are a classical machine learning approach using a large number of machine-trained decision trees. The strength of random forest comes with reduced overfitting by increasing the number of random predictive “votes”. This was useful for our smaller dataset of five or six samples per time/condition and multi-omics method. Pairing this capable method with SHAP provided a simple and explainable scoring method. SHAP uses game theory to explain individual feature contributions as a factor of model prediction. The higher the score for a feature, the more likely it is to be a predictor for a condition.

The machine learning analysis provided a streamlined understanding of the top predictive transcripts, proteins, and metabolites to identify H-ARS. Overall, dysregulation of various biological mechanisms, such as early transcriptional markers (*Sapcd2*, *Cd28* at 1 h), iron homeostasis (FRIL1), and mitotic regulation (CDK1), was shown within the first 24 h post-radiation. Machine learning analysis also identified metabolite candidate biomarkers. These included 3-hydroxyisobutyrate (3-HIB), involved in valine metabolism and a signal for fatty acid transport, at 1 h and 6 h and taurine at 24 h. The top predicted transcript, *GM47985*, at 24 h is a poorly characterized predicted protein-coding gene that may be a novel target for radiation studies in mice. Bar graphs of abundances over time for all transcripts, proteins, and metabolites listed in this section can be found in Figure S6.

### 3.7. Limitations

There are a few limitations of our study that are worth noting. First, it is important to clarify that as a result of our experimental design, any cells that died from radiation exposure were not analyzed. This survivor-biased approach may have skewed the analysis compared to results potentially obtained with a mixture of live and dead cells. In addition, the protein abundances measured in our proteomics experiment had relatively large variations at 24 h post-radiation. This is likely a result of a combination of the heterogeneous nature of DNA damage, the rates of protein dysregulation in damaged cells, and the limited sample size. These factors may have contributed to the relatively low number of statistically significant proteins observed in this study. Any overfitting of the machine learning analysis due to the limited sample size was mitigated through LOOCV, 1000-iteration permutation testing, and feature-stability tracking. The candidate biomarkers identified represent putative biomarkers that may require future experimental validation.

## 4. Materials and Methods

Analyses were performed on bone marrow tissue samples extracted from C57BL/6 male mice aged 10 weeks (*n* = 24 for proteomics; *n* = 20 tissue samples were divided for transcriptomics and metabolomics). The 5 or 6 replicate bone marrow samples were each collected following whole-body radiation exposure (5.96 Gy) at time points of 1 h post-irradiation, 6 h post-irradiation, and 24 h post-irradiation, along with samples from control individuals with no radiation exposure (No RAD). Transcriptomics sample preparation was performed by RNA extraction using the Qiagen RNeasy Plus Mini Kit (Qiagen, Hilden, Germany). Samples with a RIN (RNA Integrity Number) value of 6 and above were forwarded for sequencing through an Illumina NovaSeq platform (Illumina, Inc., San Diego, CA, USA). Proteomics analysis was performed by tissue lysis via sonication followed by in-solution trypsin digestion (Promega Corporation, Madison, WI, USA). LC-MS/MS proteomics was performed with a Thermo Scientific Orbitrap Exploris 240 coupled with a Thermo Scientific Vanquish Neo UHPLC (Thermo Fisher Scientific, Waltham, MA, USA). Metabolomics sample preparation included tissue lysis by sonication before protein precipitation with cold methanol (Fisher Chemical, Waltham, MA, USA). NMR metabolomics utilized a Bruker AVANCE III 700 MHz spectrometer (Bruker BioSpin, Billerica, MA, USA) equipped with a 5 mm quadrupole resonance QCI-P cryoprobe and a SampleJet automated sample changer with Bruker ICON-NMR software version 3.6.1. Proteomics and transcriptomics data were compared to *Mus musculus* protein and transcript libraries. Metabolite identifications were made using ChenomX 11 software (Chenomx Inc., Edmonton, AB, Canada). As appropriate, omics datasets were batch-corrected, aligned, referenced, scaled and normalized prior to univariate and multivariate statistical analyses. Biomarker analysis was performed using Random Forest and quantified using Shapley Additive exPlanations. To account for the small sample size, machine learning models were trained and evaluated using LOOCV. Model reliability and statistical significance were established using a 1000-iteration permutation test to generate empirical *p*-values. Feature stability was calculated by tracking the selection frequency of the top 20 predictive biomarkers across all cross-validation folds. See the Supporting Information Section S1 for a detailed description of the methods [58,59,60,61,62,63,64,65,66,67,68,69].

## 5. Conclusions

Our multi-omics analysis revealed a distinct temporal progression of the bone marrow response to radiation exposure, our results suggest an early defense against ferroptosis, followed by a metabolic shift resembling the Warburg effect, mitotic catastrophe associated with transcript–protein discordance, and ultimately cell death driven by apoptotic pathways and necroptosis. By focusing on the surviving cells, we gained a better understanding of bone marrow recovery mechanisms after radiation exposure and generated critical information that may be relevant to patient survival. The changing transcripts, proteins, and metabolites detected in this study may provide valuable targets for the future development of new H-ARS treatments and diagnostic tools following radiation exposure.

## Data Availability

The ELIXIR deposition databases were used as repositories for the multi-omics data used in this study. Transcriptomics RNA-seq data has been deposited via the ArrayExpress repository with the data set identifier E-MTAB-17632 [70]. The proteomics mass spectrometry data has been deposited in the ELIXIR collaborating ProteomeXchange Consortium via the PRIDE partner repository with the data set identifier PXD083967 [71]. NMR metabolomics spectra were deposited in the MetaboLights repository with the data set identifier MTBLS15571 [72]. Descriptions of this study, processed data, and links to the three depositories listed are deposited in the BioStudies repository with the repository identifier S-BSST3366 [73].

## Supplementary Materials

The supporting information can be downloaded at: https://www.mdpi.com/article/10.3390/ijms27209363/s1.
