# Supplementary material for: Temporal Multi-Omics Analysis of Mouse Bone Marrow Responses to Radiation Exposure
Source: Int J Mol Sci. 2026 Oct 21;27(20):9363. doi: 10.3390/ijms27209363 (PMC13705461; doi:10.3390/ijms27209363)
Supplement: Supplementary file 1 [file ijms-27-09363-s001.zip › ijms-4596204-supplementary/MouseBM-TemporalMultiOmics-S1.pdf]

Temporal Multi-Omics Analysis of Mouse Bone Marrow Responses to Radiation Exposure – Supporting Information 1

Ashley D. Clifton,[1,†] Micah J. Jeppesen,[1,†] Shohaib Shaffiey,[2,†] Arpita Chatterjee,[3] Kia T. Liermann-Wooldrik,[3] Oluwaseun Adebisi,[3] Simran Takkar,[3] Naresh Mutha,[4] Rebecca E. Oberley-Deegan,[3] Chittibabu Guda,[4] Massimiliano Pierobon,[2] James W. Checco,[1,5,*] and Robert Powers[1,5,*]

[1]Department of Chemistry, University of Nebraska-Lincoln, Lincoln NE 68588-0304
[2]School of Computing, University of Nebraska-Lincoln, Lincoln NE 68588-0115
[3]Department of Biochemistry and Molecular Biology, University of Nebraska Medical Center, Omaha NE 68198-5870
[4]Genetics, Cell Biology and Anatomy, University of Nebraska Medical Center, Omaha, NE 68198-5805
[5]Nebraska Center for Integrated Biomolecular Communication, University of Nebraska-Lincoln, Lincoln NE 68588-0304

Section S1 - Materials and Methods 2
Section S1.1 - Materials and Chemicals 2
Section S1.2 - Mouse handling and treatment 3
Section S1.3 - Multi-omics sample preparation 4
Section S1.3.1 - Transcriptomics sample preparation 4
Section S1.3.2 - Proteomics sample preparation 4
Section S1.3.3 - Metabolomics sample preparation 5
Section S1.4 - Multi-omics data collection 5
Section S1.4.1 - Transcriptomics data collection 5
Section S1.4.2 - Proteomics data collection 6
Section S1.4.3 - Metabolomics data collection 6
Section S1.5 - Multi-omics data analysis 7
Section S1.5.1 - Transcriptomics data analysis 7
Section S1.5.2 - Proteomics data analysis 8
Section S1.5.3 - Metabolomics data analysis 9
Section S1.6 - Integrated analysis 9
Section S1.7 - Predictive biomarker discovery 10
Section S2 - Data normalization 12
Section S3 - References 13

Figure S1 - Normalization Distributions........ 15
Figure S3 – Metabolic reprogramming selected bar graphs 17
Figure S4 – Cell-cycle suppression and mitotic catastrophe selected bar graphs........ 18
Figure S6 – Candidate biomarkers selected bar graphs 20
Tables S1-S8 21
Tables S9-S17 21
Tables S18-S25 21
Tables S26-S27 21

Section S1 - Materials and Methods

Section S1.1 - Materials and Chemicals

RNeasy plus mini kit (Qiagen 74136), Trypsin (Promega Corp. V5111), iodoacetamide (MilliporeSigma 1149-5G), dithiothreitol (MilliporeSigma 43819-5G), Rapigest-SF (Waters Corporation 186001861), urea (Sigma-Aldrich U5378-1KG), Tris base (Sigma-Aldrich 648310-500GM-M), HCl (Thermo Fisher Scientific S25838A), LC-MS grade formic acid (Thermo Fisher Scientific A117-50), Peptide Cleanup C18 spin columns (Agilent Technologies 5188-2750), Thermo Scientific Nanodrop One Spectrophotometer with WiFi and Qubit 4 Fluorometer (Thermo Fisher Scientific 13-400-525), water with 0.1% Formic Acid (v/v), Optima LC-MS grade (Fisher Scientific LS118-4), Acetonitrile with 0.1% Formic Acid (v/v), Methanol Optima LC-MS grade (Fisher Scientific A456-500), EASY Optima LC-MS grade (Fisher Scientific LS120-4), EASY-Spray UHPLC column (Thermo Scientific 75 μm X 150 mm, ES900), PepMap Neo Trap Cartridge (Thermo Scientific 174500), deuterium oxide (D, 99.9%) (Cambridge Isotope Laboratories, Inc. DLM-4-1000), TMSP-2,2,3,3-D_4 (D, 98%) sodium-3-trimethylsilylpropionate (Cambridge Isotope Laboratories, Inc. DLM-48-5X1), sodium phosphate dibasic heptahydrate (Honeywell Fluka S9390500G), sodium phosphate monobasic monohydrate (Fisher Scientific S369-500).

Section S1.2 - Mouse handling and treatment

44 C57BL/6 male mice (aged 10 weeks) were obtained from Jackson Laboratories (Bar Harbor, ME, USA). All mice were housed at the University of Nebraska Medical Center (UNMC) in accordance with the Guide for Care and Use of Laboratory Animals by the National Institutes of Health. Mice were kept on a 12-hour light/dark cycle with food and water available *ad libitum*. Animal treatment procedures were approved and enforced by the UNMC Institutional Animal Care and Use Committee (20-019-03-FC).

Male C57Bl/6 mice were humanely restrained within a 50 mL conical tube with air holes to keep the radiation plane consistent. Mice received 5.96 Gy of whole-body irradiation using a RadSource 2000 X-Ray Box Irradiator (Buford, GA, USA) with a dose rate of 1.2 Gy/minute. Mice were euthanized according to the IACUC protocol. Femurs and tibias were collected from each mouse. Bones were cleaned by removing the skin and muscular tissues. The ends of the femur and tibia were cut, and a 25 G needle with a syringe filled with HBSS (+5% FBS) was used to flush out the bone marrow. Flushing was repeated 3 times. The isolated bone marrow was passed through a cell strainer to remove any clumps of dead cells. Live, white blood cells were counted using a hemocytometer with trypan blue. Bone marrow tissues were aliquoted for analysis and snap frozen.

Mouse bone marrow tissues were aliquoted for proteomics from 24 mice. These included 6 replicate animals from each of the following time points: 1) no radiation control (No RAD), 2) 1-hour post-irradiation, 3) 6 hours post-irradiation, 4) 24 hours post-irradiation. Metabolomics and

transcriptomics samples were acquired from a second batch consisting of 20 animals with 5 replicate animals for each time point listed above. All bone marrow samples were stored at -80 °C or kept on cold blocks (-20 °C) unless otherwise stated.

Section S1.3 - Multi-omics sample preparation

Section S1.3.1 - Transcriptomics sample preparation

Total RNA was isolated from bone marrow cells using Qiagen RNeasy plus mini kit. RNA quality was assessed using RNA quality number metrics. Samples with a RIN (RNA Integrity Number) value of 6 and above were forwarded for sequencing through an Illumina NovaSeq platform.

Section S1.3.2 - Proteomics sample preparation

For proteomics, the bone marrow tissue samples were suspended in 300 µL of 2 M urea, 100 mM Tris-HCl, and 0.1% (v/v) Rapigest-SF at pH 8 and then lysed by ultra-sonication in cold blocks. The sonication was performed using a probe sonicator with variable amplitude as follows: 70% power with 3 cycles of 20 s on and 10 s off. The protein concentration of the lysates was determined by nanodrop spectrophotometry, and then 20 µg of each protein lysate was subjected to trypsin digestion (1:50 enzyme:protein) for 16 h. The reaction was quenched with 1% LC-MS grade formic acid (FA). Samples were vortexed and incubated for one hour at 4 °C to allow Rapigest-SF degradation. Samples were then subjected to solid-phase extraction and dried via a speed-vac concentrator. Samples were reconstituted in 20 µL LC-MS H_2O and 0.1% FA. Final protein concentrations were determined by nanodrop spectrophotometry and diluted to allow sample injections of 300 ng total protein. A pooled QC sample was created by mixing an equal volume of each sample.

Section S1.3.3 - Metabolomics sample preparation

Bone marrow tissue samples were suspended in 500 μL of cold methanol. Tissues were lysed by ultra-sonication in cold blocks. Sonication was performed using a probe sonicator with variable amplitude as follows: 70% power with 3 cycles of 20 s on and 10 s off. The lysate was vortexed to ensure full precipitation of proteins and then centrifuged for 20 min at 13,000 × g and 4 °C. The supernatant was transferred to a second centrifuge tube. The precipitate was resuspended in 500 μL cold 80:20 methanol:water and then centrifuged again. The process was repeated with the addition of 500 μL cold water. All supernatants were combined, snapped frozen, and dried via speed-vac. Total protein concentration of each lysate was determined by Pierce Micro BCA. Dried samples were then kept frozen at –20 °C or resuspended in 70 μL of 50 mM sodium phosphate buffer, 500 μM sodium 3-(trimethylsilyl)propionate (TMSP) in D_2O, pH 7.2. Each of the samples were then transferred into a 1.7 mm NMR tube for analysis.

Section S1.4 - Multi-omics data collection

Section S1.4.1 - Transcriptomics data collection

RNA sequencing libraries were prepared using the NuGen Universal kit with Unique Dual Indexing. Sequencing was performed on the Illumina NovaSeq platform to generate 2 × 100 bp paired end reads. Raw gene counts were obtained from sample specific *.tab* files generated following RNA-seq alignment using STAR. Quality control of sequencing and alignment metrics was performed using MultiQC.

Section S1.4.2 - Proteomics data collection

Samples were analyzed by LC-MS/MS in a randomized injection order with regular injections of the pooled QC sample and two standard samples consisting of either a trypsin digested cytochrome C or yeast proteome digest. The pooled QC and cytochrome c standards were injected after every 6 experimental proteomics samples. The standard yeast digest was the first and last sample injected in the dataset. The LC-MS/MS data were acquired with a Thermo Scientific Vanquish Neo UHPLC coupled to a Thermo Scientific Orbitrap Exploris 240. For reversed-phase separation, solvent A contained LC-MS grade H_2O and 0.1% FA. Solvent B contained 80% LC-MS grade acetonitrile in H_2O and 0.1% FA. The gradient for separation was as follows: 0 – 1 min, 1 – 3% B; 1 – 52 min, 3 – 25% B; 52 – 60 min, 25 – 40%; 60.1 – 66 min, 99% B; 66 – 67 min, 1% B. The sample was loaded onto a trap column at a flow rate of 10 µL/min for four ZebraWash cycles. Peptide separation occurred over 67 mins with 0.350 µL/min flow rate and 35 °C column temperature. For mass spectrometry, the spray voltage was set to 1900 V and ion transfer tube temperature was set to 275 °C. MS/ddMS^2 acquisition was operated in positive-ion mode utilizing higher-energy collisional dissociation. The mass spectrometry measurements were performed using the following settings: a mass range of 375-1400 *m/z*; peak intensity threshold of 5.0×10^4; full MS resolution of 60,000 and MS/MS resolution of 15,000; dynamic exclusion time was set with a 20 s window.

Section S1.4.3 - Metabolomics data collection

Samples were analyzed in random order on a Bruker AVANCE III 700 MHz spectrometer equipped with a 5 mm quadrupole resonance QCI-P cryoprobe (^{1}H, ^{13}C, ^{15}N, and ^{31}P) with a ^{2}H lock and decoupling. A SampleJet automated sample changer with Bruker ICON-NMR software

was used to automate data collection. 1D ^{1}H NMR spectra were collected using an excitation sculpting pulse program to remove the water signal. The 90-degree pulse was calculated using a representative sample and applied across all samples. Locking, shimming, tuning, and matching were done manually for the first sample and then applied programmatically through the ICON automation software. Each spectrum was collected at 298 K with a sweep width of 9124 Hz, 64 K data points, 4 dummy scans, and 128 scans. The TMSP signal was used as a reference to align chemical shifts across experiments.

Section S1.5 - Multi-omics data analysis

Section S1.5.1 - Transcriptomics data analysis

Sample specific STAR gene count files were merged by gene identifier to construct a unified gene by sample count matrix for each time point. These matrices were used as input for differential expression analysis using PyDESeq2 library in Python.[1] The DESeq2 model, which is based on a negative binomial distribution, was applied to normalize counts and perform statistical testing (Wald test) to identify differentially expressed genes. PyDESeq2 used in this study is the Python implementation of the DESeq2 model. Raw *p*-values were adjusted for multiple testing using the Benjamini-Hochberg procedure. Genes with an adjusted *p*-value (p_{adj}) < 0.05 and an absolute $\log_2$ fold change (Log_2FC) > 1.0 were considered significantly differentially expressed/abundant. Multivariate analysis was performed using principal component analysis (PCA) to observe unsupervised separation between control and irradiated groups.[2]

Section S1.5.2 - Proteomics data analysis

Proteomics data was analyzed using PEAKS Studio 11, searching against the *Mus musculus* Swiss-Prot database from UniProt. LC-MS/MS files were processed first using label-free quantification in PEAKS Studio 11, and then followed by a statistical analysis using MVAPACK (https://mvapack.unl.edu/).[3] For PEAKS Studio 11 processing, the *de novo* parameters were: precursor mass error tolerance: 10.00 ppm, fragment mass error tolerance: 0.05 Da, enzyme: trypsin, max variable PTM per peptide: 3, fixed modifications: carbamidomethylation (+57.02), variable modifications: oxidation (M) (+15.99). The database search parameters additionally included: digest mode: specific, peptide length range: 6 – 45, deep learning boost: enabled. The following were filters for database reporting: PSM FDR: 1.0%, proteins –10LogP ≥ 15.0, proteins unique peptides ≥ 1, *de novo* only ALC ≥ 50.0%, *de novo* only tab sharing: disabled, *de novo* only fully matched: no. Label free quantification (LFQ) parameters were: LFQ method: identification directed quantification, mass error tolerance: 20.00 ppm, retention time shift tolerance: 2.0 min, feature intensity ≥ 5000.0, retention time range: 0.0 - max, and base sample used was a pooled QC. The LFQ feature filters: average area ≥ 0.0, Quality ≥ 20.0, charge: 2 – 8, peptide ID count ≥ 1 per group, detected in at least one pooled QC and one sample, and the in-group coefficient of variation filter was not used. The LFQ protein filters were: significance method: ANOVA, modified form exclusion: yes, remove outlier was not done, used: top 3 peptides, significance filter: 0, and each protein had at least 1 peptide. Total ion current was used to normalize each spectrum. LFQ protein data was exported and further processed in MVAPACK. Prior to statistical analysis, missing values within the abundance data were imputed using the minimum abundance level per protein among the rest of the samples.[4] A log transformation was also applied prior to further analysis. Batch correction was performed using the pooled QC data based on robust LOESS signal correction.[5] Following batch correction, a normalization step was performed using the median. Statistical

analyses were performed using Student's *t*-test, fold-change analysis, hierarchical clustering, and PCA. Raw *p*-values were adjusted for multiple hypotheses using the Benjamini-Hochberg procedure. Proteins with an adjusted *p*-value (p_{adj}) < 0.05 and an absolute $\log_2$ fold change (Log_2FC) > 1.0 were considered significantly increased or decreased.

Section S1.5.3 - Metabolomics data analysis

Metabolite identifications were made using ChenomX 11 software. Subsequent statistical analysis was performed using MVAPACK.[3] The raw data was processed in MVAPACK by adding one zero fill, apodizing to an exponential window, and phasing using the automated routine. Fourier transformation was done using MVAPACK's built-in functions. The adaptive binning routine was used, and the product bins were normalized by standard normal variate.[6] For ChenomX 11 processing, raw data was loaded using TMSP as the internal standard. A baseline correction was performed using the ChenomX spline method. Metabolites were matched first manually using a representative sample and then automatically added to each spectrum. Manual inspection followed to verify fit. ChenomX 11 data was exported as peak areas and normalized by median prior to statistical modeling. Statistical analyses were performed using Student's *t*-test, fold-change analysis, hierarchical clustering, and PCA. Raw *p*-values were adjusted for multiple hypotheses using the Benjamini-Hochberg procedure. Metabolites with p_{adj} < 0.05 and an absolute Log_2FC > 1.0 were considered significantly increased or decreased.

Section S1.6 - Integrated analysis

For downstream pathway and functional analyses, a unified molecular signature was generated at each time point by combining the lists of significant differentially expressed genes and

differentially expressed proteins ($p_{adj} < 0.05$ and absolute $Log_2FC > 1$). Gene/protein identifiers were standardized, and duplicate entries were resolved by averaging their Log_2FC values.

Pathway enrichment analysis was conducted on the significant up- and down-regulated molecules ($p_{adj} < 0.05$ and absolute $Log_2FC > 1$). within the unified signatures for each time point. The analysis was performed using the g:Profiler web tool (*Mus musculus*), querying the Gene Ontology (GO), KEGG, and Reactome databases. Terms with an adjusted *p*-value (p_{adj}) < 0.05 were considered significantly enriched.[7]

To evaluate the temporal response after radiation exposure, differentially expressed genes, proteins, and metabolites were integrated across the 1, 6, and 24-hour timepoints. The top four biological responses categories were: ferroptosis defense, cell death, Warburg effect, and mitotic catastrophe. This categorization was accomplished by manually cross-referencing functional pathway annotations (derived from g:profiler, KEGG, and Metaboanalyst) with established signs of radiation damage. A unified matrix was then constructed to compare Log_2 fold-changes across the transcripts, proteins, and metabolites. Discordance analysis was performed to identify discrepancies between transcript expression levels and the abundance of their corresponding proteins (e.g., a negative transcript Log_2FC alongside a positive protein Log_2FC).

Section S1.7 - Predictive biomarker discovery

To identify molecules that are most predictive of radiation exposure, parallel machine learning models were developed for transcriptomic, proteomic, and metabolomic datasets. For each data

type and time point, a Random Forest Classifier was trained on normalized abundance data (gene counts, protein intensity, metabolite abundance) to distinguish between irradiated and control samples using the Scikit-learn library in Python.[8] Training was conducted using Leave-One-Out Cross-Validation (LOOCV). Overall model significance was validated against a null distribution generated by a 1000-iteration permutation test. The contribution of each gene, protein, and metabolite to the model's predictive accuracy was quantified using SHapley Additive exPlanations (SHAP).[9] Feature stability was calculated by how often a molecule was selected among the top 20 predictors across all cross-validation folds, expressed as a percentage. To prioritize the most reliable biomarkers, the final candidates were ranked primarily by this stability percentage, using the average SHAP score as a tie-breaker for molecules with equal stability.

Section S2 - Data normalization

Large changes in metabolite and protein abundances were observed when comparing No RAD and 24 hours post-irradiation. These large changes caused a skew in protein and metabolite abundance distributions. A shift in the abundance density forced an evaluation of normalization strategies. Two traditional approaches, median and sum normalization, were compared by output densities. The distributions of these normalization outcomes and raw values are shown in Figure S1.4. Traditional normalization strategies hold a critical assumption that there is a small number of variables that have the majority of the variability in the dataset. The median normalization method was observed to be more effective in aligning the median, the first, and the second quartiles of each group. Median normalization is a common technique in -omics studies and aims to align the median values of samples.[10] Recent comparisons of normalization strategies in temporal multi-omics studies have found median normalization to be well suited to preserve natural variation.[11] The difference between both normalization strategies was most prevalent in the proteomics data. The sum normalization of the proteomics data was observed to be distorted by a high abundance skewness. The metabolomics dataset was also affected by a high abundance skewness, as well as a larger low abundance skewness. As previously demonstrated, the proper choice of data analysis method is critical to achieve reliable and accurate results from any -omics or multi-omics study.[12]

Section S3 - References

(58) Love, M. I.; Huber, W.; Anders, S. Moderated estimation of fold change and dispersion for RNA-seq data with DESeq2. *Genome Biology* **2014**, *15* (12), 550. DOI: 10.1186/s13059-014-0550-8.

(59) Benjamini, Y.; Hochberg, Y. Controlling the False Discovery Rate: A Practical and Powerful Approach to Multiple Testing. *Journal of the Royal Statistical Society: Series B (Methodological)* **2018**, *57* (1), 289-300. DOI: 10.1111/j.2517-6161.1995.tb02031.x.

(60) Jurich, C. P.; Jeppesen, M. J.; Sakallioglu, I. T.; De Lima Leite, A.; Yesselman, J. D.; Powers, R. Simulated LC–MS Data Set for Assessing the Metabolomics Data Processing Pipeline Implemented into MVAPACK. *Analytical Chemistry* **2024**, *96* (32), 12943-12956. DOI: 10.1021/acs.analchem.3c04979.

(61) Pedregosa, F.; Varoquaux, G.; Gramfort, A.; Michel, V.; Thirion, B.; Grisel, O.; Blondel, M.; Prettenhofer, P.; Weiss, R.; Dubourg, V.; et al. Scikit-learn: Machine Learning in Python. *J. Mach. Learn. Res.* **2011**, *12* (null), 2825–2830.

(62) Dunn, W. B.; Broadhurst, D.; Begley, P.; Zelena, E.; Francis-McIntyre, S.; Anderson, N.; Brown, M.; Knowles, J. D.; Halsall, A.; Haselden, J. N.; et al. Procedures for large-scale metabolic profiling of serum and plasma using gas chromatography and liquid chromatography coupled to mass spectrometry. *Nature Protocols* **2011**, *6* (7), 1060-1083. DOI: 10.1038/nprot.2011.335.

(63) Worley, B.; Powers, R. Generalized adaptive intelligent binning of multiway data. *Chemometrics and Intelligent Laboratory Systems* **2015**, *146*, 42-46. DOI: https://doi.org/10.1016/j.chemolab.2015.05.005.

(64) Raudvere, U.; Kolberg, L.; Kuzmin, I.; Arak, T.; Adler, P.; Peterson, H.; Vilo, J. g:Profiler: a web server for functional enrichment analysis and conversions of gene lists (2019 update). *Nucleic Acids Research* **2019**, *47* (W1), W191-W198. DOI: 10.1093/nar/gkz369.

(65) Breiman, L. Random Forests. *Machine Learning* **2001**, *45* (1), 5-32. DOI: 10.1023/A:1010933404324.

(66) Lundberg, S. M.; Lee, S.-I. A unified approach to interpreting model predictions. In Proceedings of the 31st International Conference on Neural Information Processing Systems, Long Beach, California, USA; 2017.

(67) Callister, S. J.; Barry, R. C.; Adkins, J. N.; Johnson, E. T.; Qian, W.-j.; Webb-Robertson, B.-J. M.; Smith, R. D.; Lipton, M. S. Normalization Approaches for Removing Systematic Biases Associated with Mass Spectrometry and Label-Free Proteomics. *Journal of Proteome Research* **2006**, *5* (2), 277-286. DOI: 10.1021/pr050300l.

(68) Tseng, C. Y.; Salguero, J. A.; Breidenbach, J. D.; Solomon, E.; Sanders, C. K.; Harvey, T.; Thornhill, M. G.; Palmisano, S. J.; Sasiene, Z. J.; Blackwell, B. R.; et al. Evaluation of normalization strategies for mass spectrometry-based multi-omics datasets. *Metabolomics* **2025**, *21* (4), 98. DOI: 10.1007/s11306-025-02297-1.

(69) Vu, T.; Riekeberg, E.; Qiu, Y.; Powers, R. Comparing normalization methods and the impact of noise. *Metabolomics* **2018**, *14* (8), 108. DOI: 10.1007/s11306-018-1400-6.

Figure S1 - Normalization Distributions

Violin plots of the group averaged all variable distributions for each –omics analysis. Displayed are the results of different normalization methods with a direct comparison to the raw distributions. The top row is the raw average distribution at each time point. The second row is the average distribution of each time point after median normalization. The third row is the average distribution of each time point after the alternate normalization method (sum normalized for metabolomics and proteomics).

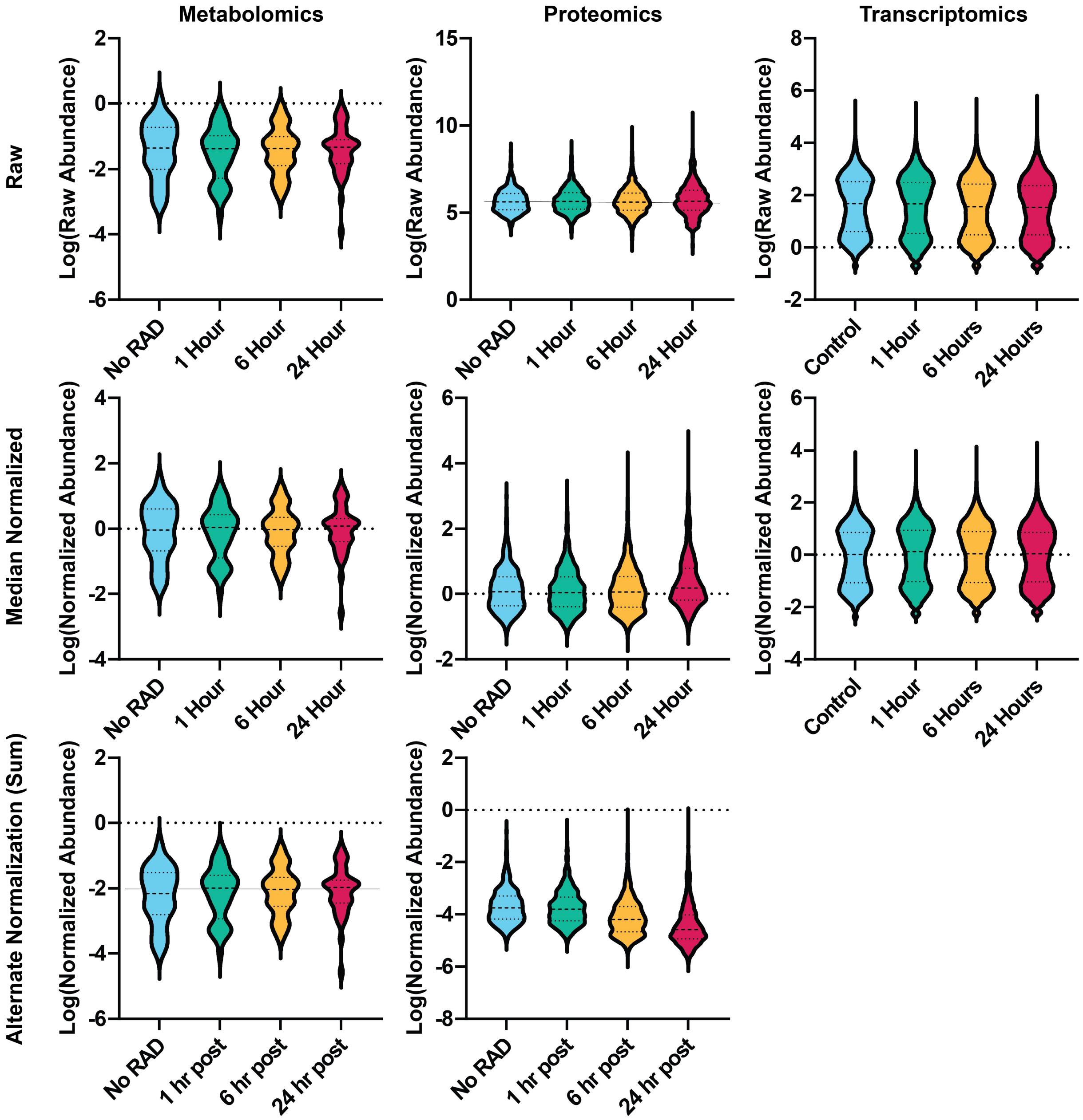

Figure S2 – Early oxidative stress and ferroptosis-defense response selected bar graphs

Bar graphs of selected genes, proteins, and metabolites. The range of normalized abundances is listed in [Min:Max] format. Ferritin light chain 1: *Ftl1* [53.7:160.9], FRIL1 [0.6:50.2]; Glutathione peroxidase 1: *Gpx1* [11.9:50.2], GPX1 [2.2:383.8]; Glutathione peroxidase 4: *Gpx4* [42.2:78.8], GPX4 [0.03:24.39]; Superoxide dismutase 1: *Sod1* [41.2:90.5], SOD1 [9.8:333.2]; histidine [0.0:1.0]; taurine [13.7:35.1]. The significance was found using Student's *t*-test and a Benjamini-Hochberg FDR correction was applied. Significance is denoted in levels as follows: * $p_{adj} < 0.05$, ** $p_{adj} < 0.01$, *** $p_{adj} < 0.001$, **** $p_{adj} < 0.0001$.

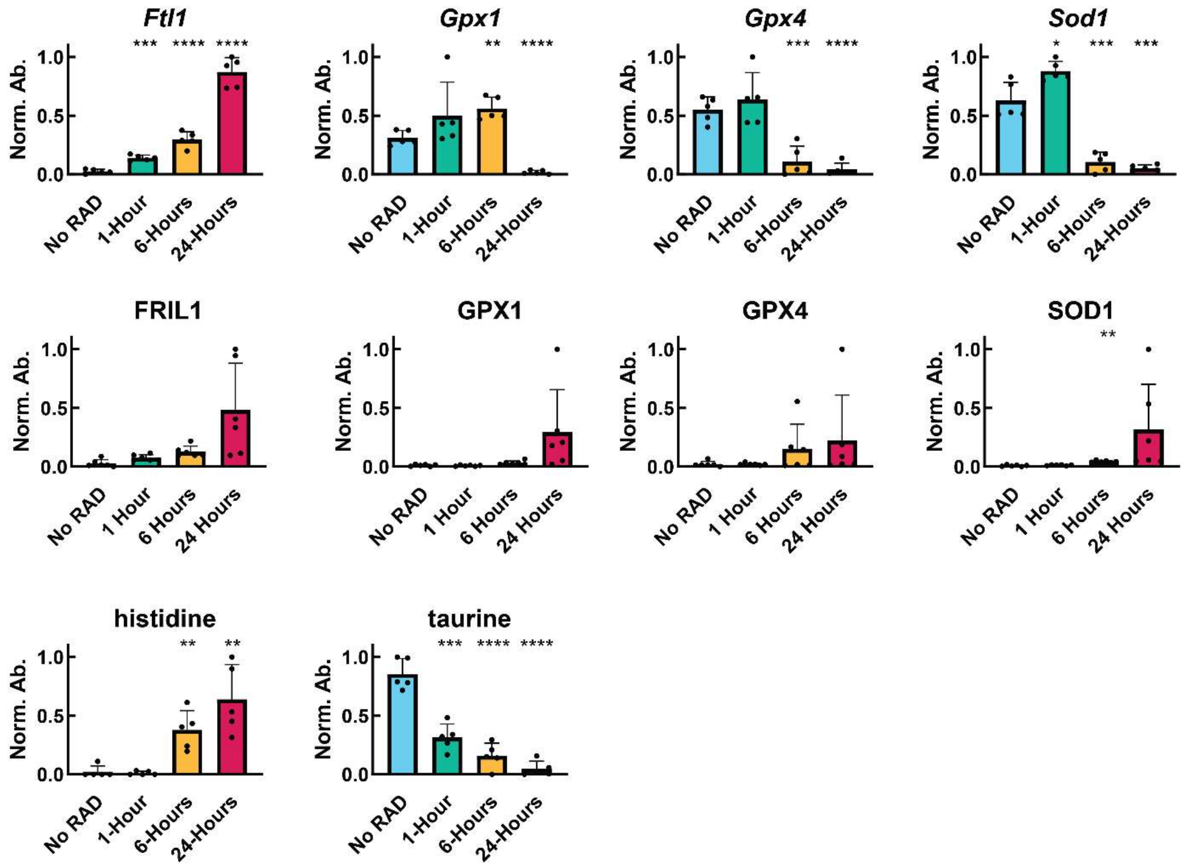

Figure S3 – Metabolic reprogramming selected bar graphs

Bar graphs of selected genes, proteins, and metabolites. The range of normalized abundances is listed in [Min:Max] format. Creatine kinase mitochondrial 1: *Ckmt1* [0.0:0.8]; Hexokinase 1: *Hk1* [62.9:106.4], HK1 [0.08:388.58]; Hexokinase 2: *Hk2* [12.5:42.8]; Pyruvate kinase: *Pkm* [76.6:230.8], PKM [9.04:128.12]; Hypoxia-inducible factor 1 subunit alpha: *Hif1a* [7.7:20.8]; malonate [0.003:0.237]; 3-hydroxyisobutyrate (3-HIB) [0.002:0.119]; adenosine di-phosphate (ADP) [0.57:3.71]; adenosine mono-phosphate (AMP) [0.86:4.38]; adenosine tri-phosphate (ATP) [0.21:3.69]. The significance was found using Student's *t*-test and a Benjamini-Hochberg FDR correction was applied. Significance is denoted in levels as follows: * $p_{adj} < 0.05$, ** $p_{adj} < 0.01$, *** $p_{adj} < 0.001$, **** $p_{adj} < 0.0001$.

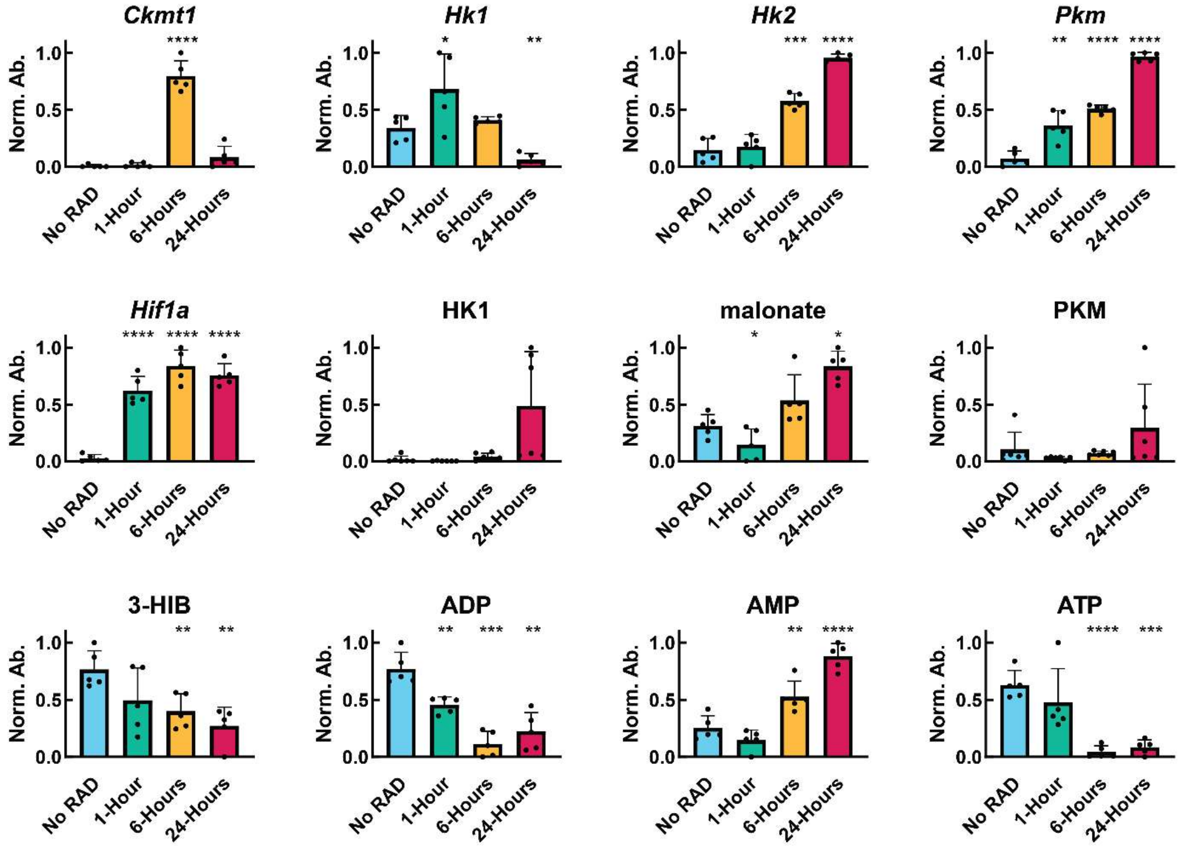

Figure S4 – Cell-cycle suppression and mitotic catastrophe selected bar graphs

Bar graphs of selected genes, proteins, and metabolites. Normalized abundance includes a min-max scaling from 0 to 1. The range of normalized abundances is listed in [Min:Max] format. Cyclin dependent kinase 1: *Cdk1* [16.5:45.5], CDK1 [0.05:0.70]; Cyclin dependent kinase 2: *Cdk2* [9.5:19.3]; Cyclin dependent kinase 4: *Cdk4* [13.5:42.1]; E2F transcription factor 1: *E2f1* [6.2:23.8]; Histone 1 cluster 1: *H1f1* [0:0.2], H1-1 [0.3:31.7]; Histone 2A: *H2ax* [11.7:76.3], H2AX [0.02:3.36]; Histone 3 cluster 2: *H3c2* [0.0:0.1], H3C2 [20.5:937.9]; Lamin B1: *Lmnb1* [53.0:146.1], LMNB1 [3.8:1649.2]; Regulator of chromosome condensation 2: *Rcc2* [35.0:93.2], RCC2 [0.4:967.0]; Structural maintenance of chromosomes 2: *Smc2* [17.7:60.4], SMC2 [0.05:8.31]; Tumor protein p53: *Trp53* [16.0:47.8]; Cyclin B2: *Ccnb2* [23.0:49.4]; Elongation factor 2: EF2 [1.0:21.6]. The significance was found using Student's *t*-test and a Benjamini-Hochberg FDR correction was applied. Significance is denoted in levels as follows: * $p_{adj} < 0.05$, ** $p_{adj} < 0.01$, *** $p_{adj} < 0.001$, **** $p_{adj} < 0.0001$.

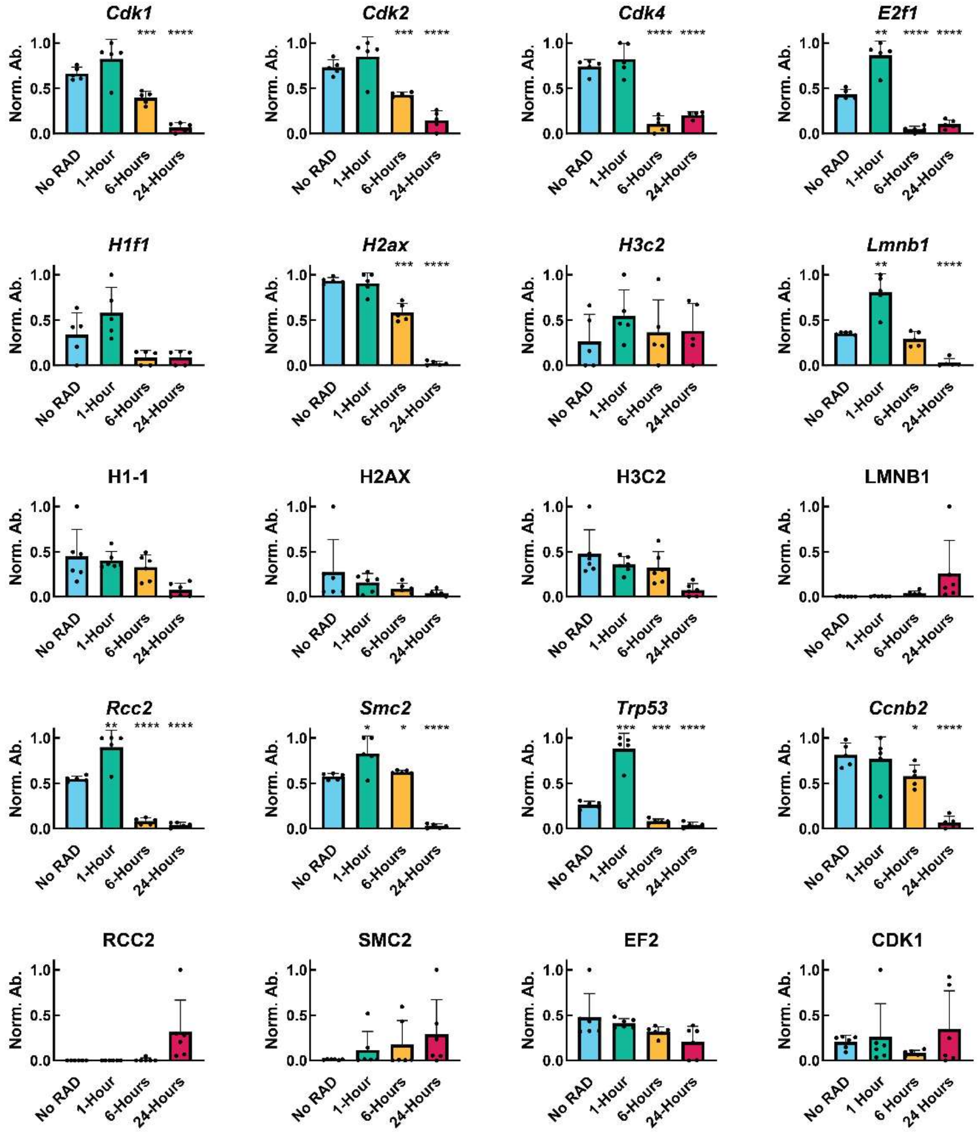

S5 – Programmed cell death selected bar graphs

Bar graphs of selected genes, proteins, and metabolites. The range of normalized abundances is listed in [Min:Max] format. BCL2 Associated X, apoptosis regulator: *Bax* [3.6:22.7]; BCL2 apoptosis regulator: *Bcl2* [4.3:10.2]; Caspase 3: *Casp3* [19.2:30.3]; Caspase 4: *Casp4* [2.46:10.2]; Caspase 6: *Casp6* [1.3:3.76], CASP6 [0.03:5.14]; Caspase 7: *Casp7* [2.9:7.2]; Caspase 8: *Casp8* [9.1:19.5]; Cytochrome C1: *Cyc1* [11.7:24.3]; MCL1 apoptosis regulator, BCL2 family member: *Mcl1* [104.4:210.5]; Fas cell surface death receptor: *Fas* [2.8:7.7], FAS [0.07:1.33]; Mixed lineage kinase domain like pseudokinase: *Mlkl* [2.0:6.5]; Peptidylprolyl isomerase F: *Ppif* [3.2:7.1]; Receptor interacting serine/threonine kinase 1: *Ripk1* [9.8:23.7]; Receptor interacting serine/threonine kinase 3: *Ripk3* [3.8:9.1]; Tumor necrosis factor: *Tnf* [1.9:4.1]; TNF receptor superfamily member 1A: *Tnfrsf1a* [27.2:81.7]; Programmed cell death 6: PDCD6 [0.1:2.0]; adenosine tri-phosphate (ATP) [0.21:3.69]; o-phosphocholine [1.0:6.1]. The significance was found using Student's *t*-test and a Benjamini-Hochberg FDR correction was applied. Significance is denoted in levels as follows: * $p_{adj} < 0.05$, ** $p_{adj} < 0.01$, *** $p_{adj} < 0.001$, **** $p_{adj} < 0.0001$.

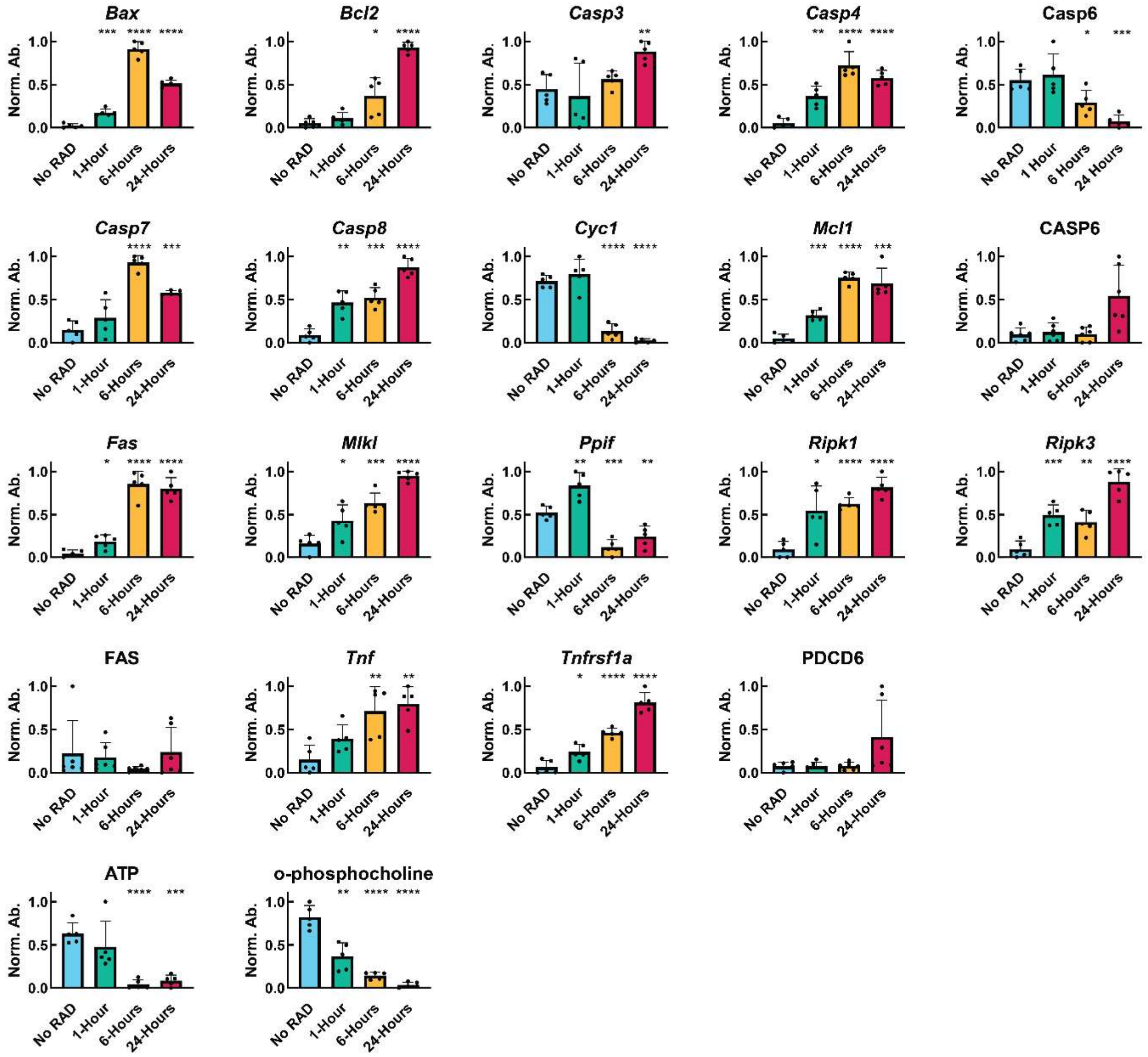

Figure S6 – Candidate biomarkers selected bar graphs

Bar graphs of selected genes, proteins, and metabolites. The range of normalized abundances is listed in [Min:Max] format. Suppressor APC Domain Containing 2: *Sapcd2* [0.67:3.40]; *C*d28 [0.4:1.5]; Ferritin light chain 1: FRIL1 [0.6:50.2]; Cyclin dependent kinase 1: CDK1 [0.05:0.70]; 3-hydroxyisobutyrate (3-HIB) [0.002:0.119]; taurine [13.7:35.1]. The significance was found using Student's *t*-test and a Benjamini-Hochberg FDR correction was applied. Significance is denoted in levels as follows: * $p_{adj} < 0.05$, ** $p_{adj} < 0.01$, *** $p_{adj} < 0.001$, **** $p_{adj} < 0.0001$.

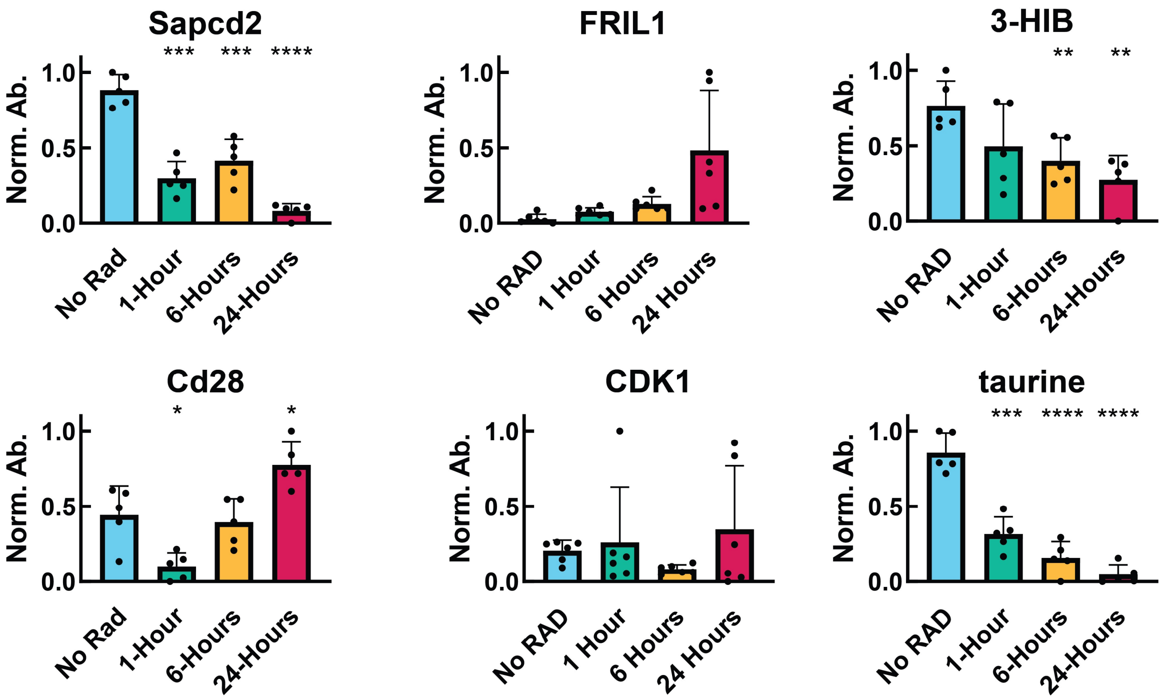

Tables S1-S8

See Transcriptomics-S2 file

Tables S9-S17

See Proteomics-S3 file

Tables S18-S25

See Metabolomics-S4 file

Tables S26-S27

See Integrated-Analysis-S5 file
